# DNA methylation as a predictor of pituitary neuroendocrine tumour behaviour: A systematic review

**DOI:** 10.1111/jne.70167

**Published:** 2026-03-22

**Authors:** Romy van der Groef, Eskeatnaf Mulugeta, Sebastian Neggers, Julie Refardt

**Affiliations:** ^1^ Erasmus MC Department of Internal Medicine Section Endocrinology Rotterdam The Netherlands; ^2^ Pituitary Centre Rotterdam University Medical Centre Rotterdam Rotterdam The Netherlands

**Keywords:** DNA methylation, epigenomics, pituitary neuroendocrine tumour, tumour behaviour

## Abstract

Pituitary neuroendocrine tumours (PitNETs) range from slow‐growing to highly aggressive tumours; however, traditional prognostic markers often fail to predict clinical outcomes reliably. DNA methylation has recently emerged as a promising biomarker for assessing tumour behaviour. This systematic review evaluates its predictive value in PitNETs. To systematically assess the clinical applicability of DNA methylation profiles in predicting behaviour of PitNETs. Systematic review. A comprehensive search was conducted in Medline, Embase, Web of Science, and Cochrane CENTRAL on December 13, 2024, with an update on October 17, 2025. The search included studies on adult PitNET patients, specifically examining tumour behaviour in relation to DNA methylation. Excluded were studies that focused on cell‐free DNA, investigated a single gene with no established relevance to tumour behaviour, or assessed tumour size only. Data were extracted from 20 eligible studies by four independent reviewers. The risk of bias was assessed using the QUIPS tool. Due to methodological differences across studies, the findings were summarised narratively. Twelve studies investigated tumour invasiveness, two examined tumour aggressiveness and five examined PitNET regrowth, recurrence and re‐intervention. The majority of studies concentrated on non‐functioning PitNETs and used Illumina arrays or PCR‐based methods. These analyses identified several differentially methylated genes linked to invasiveness *(*e.g., *PHYHD1*, *WNT4*, *STAT6*, *CDH1*, *CDH13*), aggressive behaviour (e.g., *AIP, PDCD1, LINE‐1*), and tumour regrowth (e.g., *TERT*, *FAM90A1*, *ING2*). DNA methylation profiling shows potential for predicting PitNET behaviour, but methodological inconsistencies limit its clinical application. Standardized methods and prospective validation are needed for clinical integration.

## 1 INTRODUCTION

Pituitary neuroendocrine tumours (PitNETs), also known as pituitary adenomas, are the most common tumours originating from the pituitary gland, accounting for approximately 10%–15% of all intracranial neoplasms.^1^ Despite their generally benign nature, PitNETs exhibit considerable clinical heterogeneity, with significant variation in their behaviour and aggressiveness. While some tumours remain indolent and asymptomatic, others can display more aggressive or invasive characteristics.^2^

Aggressive PitNETs are often marked by increased cellular proliferation and frequently show poor responses to conventional therapies, such as surgery, radiotherapy, and medical treatment.^2^ Recent guidelines from the European Society of Endocrinology recommend considering a diagnosis of aggressive PitNETs in patients with radiologically invasive tumours that exhibit rapid growth or clinically relevant progression despite optimal therapies.^2^ This definition, however, only captures a small subset of PitNETs with the most aggressive clinical course. Other efforts to define tumour behaviour have incorporated a wide range of outcomes, such as invasiveness, aggressiveness, tumour recurrence, progression‐free survival, and the need for re‐intervention.^3^, ^4^, ^5^, ^6^, ^7^, ^8^, ^9^, ^10^, ^11^, ^12^, ^13^, ^14^, ^15^, ^16^, ^17^, ^18^, ^19^, ^20^, ^21^ This variability complicates the consistent identification of aggressive PitNETs.

**FIGURE 1:**
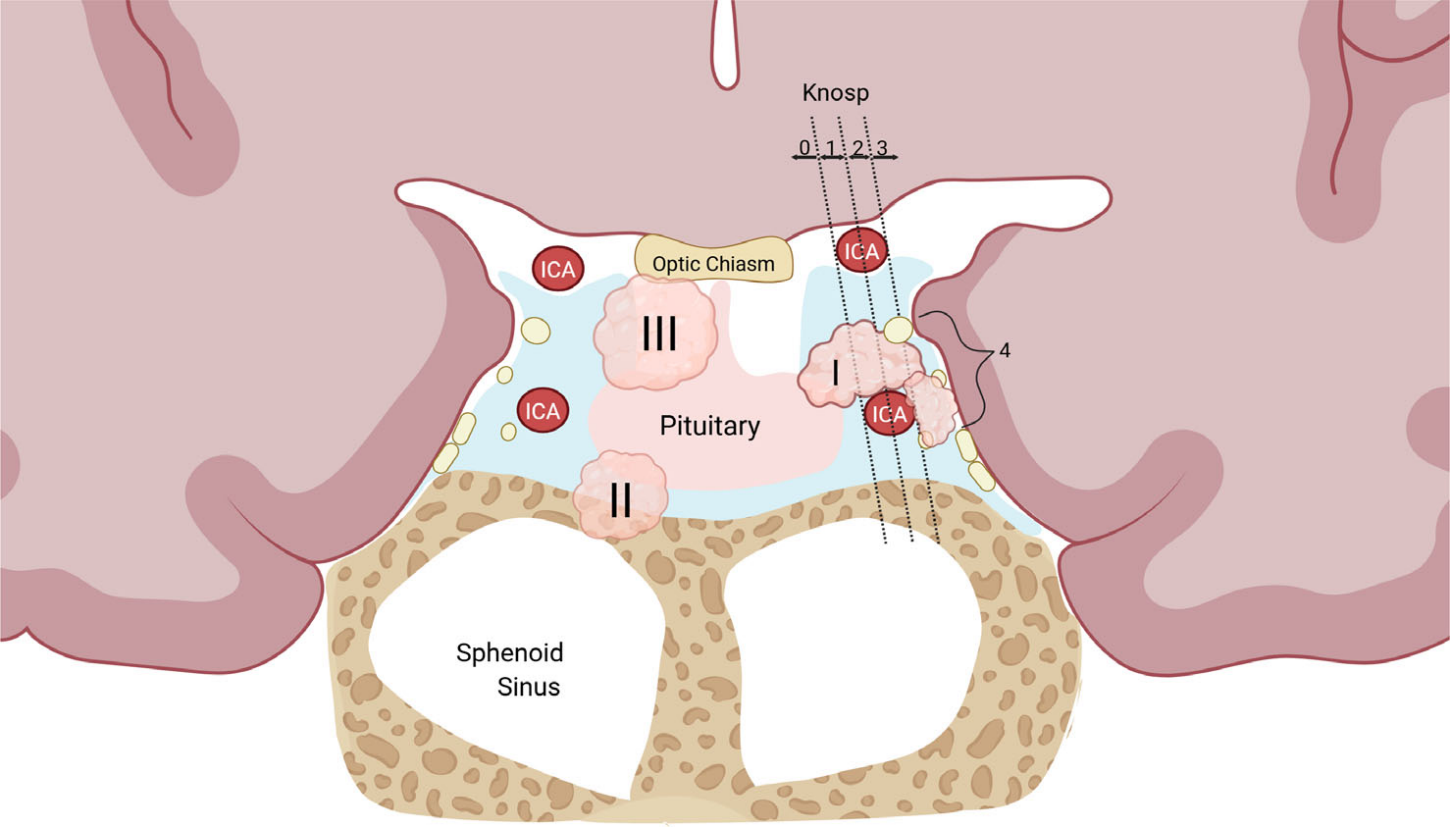
Pituitary tumour invasion. Anatomical illustration of the suprasellar and sellar turcica region, highlighting key landmarks relevant to evaluating PitNET cavernous sinus invasion. PitNET invasion can be classified as parasellar (I), infrasellar (II), or suprasellar (III). Parasellar invasion is further categorized using the modified Knosp and Hardy‐Wilson classification systems. As shown, Knosp Grade 3 indicates tumour extension up to the lateral tangent of the cavernous sinus, while Grade 4 represents complete encasement of the intracavernous internal carotid artery. In the Hardy‐Wilson system, stage D corresponds to intracranial extension, and stage E indicates cavernous sinus extension. Created in BioRender. Van der Groef, R (2026) https://BioRender.com/k720amz.

Invasive PitNETs infiltrate surrounding anatomical structures, such as the cavernous sinus and sphenoid sinus, complicating surgical resection and thereby negatively impacting patient outcomes.^22^, ^23^ Tumour invasiveness is typically assessed using established staging systems, including the adapted Knosp grades 3–4 and Hardy–Wilson stages D–E, which provide a standardized approach to evaluating tumour invasion (Figure 1).^1^, ^22^, ^24^, ^25^

Predicting the invasive or aggressive behaviour of PitNETs remains a clinical challenge. The Ki67 proliferative index, commonly used as a biomarker of tumour proliferation,^26^ is often associated with aggressive behaviour and invasiveness in PitNETs. However, its prognostic value remains limited, as a low Ki67 index does not necessarily exclude the possibility of aggressive behaviour, nor does a high Ki67 index guarantee it.^23^

In recent years, the role of epigenetic modifications, particularly DNA methylation, in the pathogenesis and clinical behaviour of PitNETs is emerging.^27^ DNA methylation typically silences gene expression through two main mechanisms: first, by directly blocking transcription factors (TF) from binding to gene promoter regions, and second, by recruiting methyl‐CpG‐binding domain (MBD) proteins. These MBD proteins then attract enzymes such as histone deacetylases and other chromatin‐remodelling complexes, which condense the chromatin structure, resulting in inactivation of the gene or reducing its transcription.^27^, ^28^, ^29^ DNA methylation alterations have been extensively studied in various cancers. Hypomethylation has been shown to lead to the overexpression of proto‐oncogenes and growth factors, which contribute to uncontrolled cellular growth and metastasis.^30^, ^31^ On the other hand, hypermethylation can lead to the silencing of tumour suppressor genes, such as TP53, thereby promoting uncontrolled cell growth.^30^, ^31^ In PitNETs, previous research has identified DNA hypermethylation of three tumour suppressor genes (*GADD45Υ*, *CDKN2A* and *MEG3*). Silencing of these genes by DNA hypermethylation stimulates cell growth and contributes to tumour development.^32^, ^33^, ^34^, ^35^ DNA methylation profiling has also emerged as a promising tool for refining tumour classification beyond traditional histopathology, enabling more accurate identification of tumour subtypes and lineage differentiation.^36^, ^37^, ^38^

While recent reviews have broadly discussed DNA methylation in PitNETs,^39^, ^40^ none have systematically focused on its clinical predictive value for aggressive and invasive tumour behaviour. Given the variability in defining these tumour behaviours, a systematic review is needed to evaluate the potential of DNA methylation as a predictive biomarker and to improve the identification of high‐risk PitNETs.

## 2 METHODS

### 2.1 Search strategy

A comprehensive systematic search of English literature was conducted on the 13 December 2024 and updated on the 17 October 2025 for studies published until October 2025. The following databases were searched: Medline ALL, Embase, Web of Science Core Collection, and the Cochrane Central Register of Controlled Trials, in order to identify relevant evidence addressing the research questions. A detailed search strategy, including keywords and Boolean operators, is provided in Appendix S1.

### 2.2 Eligibility criteria and study selection

Studies were independently screened by two reviewers (RvdG and JR) using Covidence.^41^ Any discrepancies between the two reviewers were resolved through consensus with a third and fourth reviewer (EM, SN). The inclusion criteria were as follows:

- Studies involving adult patients diagnosed with a PitNET.
- Studies that assessed tumour behaviour with DNA methylation analysis.

Exclusion criteria included:

- Studies performing DNA methylation analysis on cell‐free DNA.
- Studies focus on the methylation status of one specific gene without established biological function in tumour behaviour.
- Studies focusing solely on tumour size.
- Studies that lacked clear definitions of tumour behaviour, or that did not assess these factors in relation to DNA methylation.

A complete overview of all in‐ and exclusion criteria is presented in Appendix S2.

### 2.3 Data extraction

Data were summarized in evidence tables, with predefined categories including: study design, treatment era, duration of follow‐up, participant characteristics, DNA methylation analysis methods, primary outcomes, and additional remarks (see Table S3 for detailed data extraction items). The data were organized to enable meaningful comparisons and synthesis of findings.

### 2.4 Assessment of risk of bias of individual studies

The risk of bias for each included study was assessed using the Quality in Prognosis Studies (QUIPS) tool,^42^ which evaluates the risk of bias across six domains: (1) study participation, (2) study attrition, (3) prognostic factor measurement, (4) outcome measurement, (5) study confounding, and (6) statistical analysis and reporting. The risk of bias was categorized as low, moderate, or high for each domain. Studies were classified as high quality if they had low risk of bias across all domains, moderate quality if they had one or more domains with moderate risk, and low quality if they had high risk of bias in any domain. To facilitate interpretation, colour coding was used to represent the quality of each study: red for low quality, orange for moderate quality, and green for high quality. A summary of the risk of bias assessment for each study is provided in Table S4.

## 3 RESULTS

**FIGURE 2:**
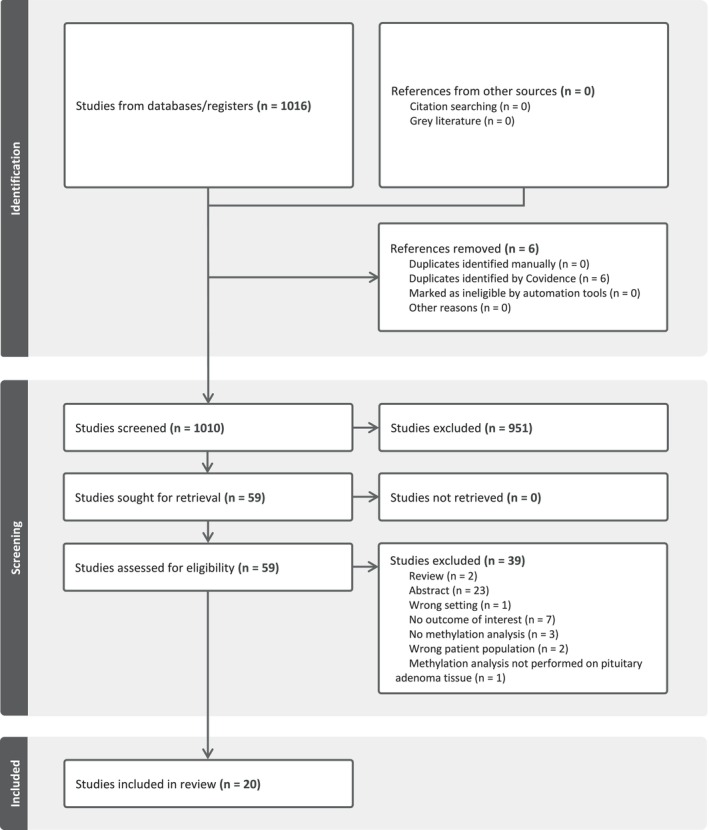
Flowchart.

Our search identified 1016 records (see Figure 2). After screening titles and abstracts, 59 full‐text articles were assessed, of which 20 met the inclusion criteria. Among these, 13 studies focused on PitNET invasiveness, two examined PitNET aggressiveness, and five investigated PitNET regrowth, recurrence, and re‐intervention. A detailed overview of the included studies is provided in Table S3.

### 3.1 Quality assessment

Quality assessment using the QUIPS tool classified 11 studies as high quality and eight as moderate quality. One study, Yang et al.^43^ received a low‐quality rating and was excluded from further discussion in this section. The low rating was due to a high risk of attrition bias, confounding bias, and issues with statistical analysis and reporting. Further details on the QUIPS quality assessment can be found in Table S4.

### 3.2 PitNET invasiveness

The relationship between DNA methylation and PitNET invasiveness has been explored in 12 case–control studies. Five of these studies^4^, ^5^, ^7^, ^12^, ^14^ focused on genome‐wide methylation profiling, primarily in non‐functioning PitNET subtypes, using techniques such as Illumina Infinium MethylationEPIC BeadChip arrays or reduced representation bisulphite sequencing (RRBS), with sample sizes ranging from 19 to 68 patients. The remaining seven studies^6^, ^11^, ^17^, ^18^, ^19^, ^20^, ^21^ focused on targeted gene methylation in both functioning and non‐functioning PitNETs, analyzing cohorts from 32 to 102 participants. These studies used methods such as DNA pyrosequencing, methylation‐specific PCR (MS‐PCR), and methylation‐specific multiplex ligation‐dependent probe amplification (MS‐MLPA).

In most studies, tumour invasiveness was defined using MRI‐based criteria (modified Knosp grades 3–4 or Hardy–Wilson grades D–E) and/or histological or intraoperative evidence of invasion into surrounding tissues, including the cavernous sinus, bone, dura, sphenoid sinus, and clivus. Tables 1 and 2 summarize study characteristics and gene‐level findings.

**TABLE 1:** Overview of studies assessing PitNET behaviour.

Study	Methylation method	Sample size (*n*)	Control group	PitNET type	Outcome(s)	Definition of outcome(s)	Main outcomes
Chen et al.	RRBS	41	Non‐invasive PitNETs	All subtypes, mainly non‐functioning	Invasiveness	Histological, radiological or surgical evidence of tumour invasion into the cavernous sinus or sphenoid sinus.	Seven genes (*FGFR3*, *KCNK2*, *C4orf50*, *SSC5D*, *FBXO2*, *SREBF1*, *SLITRK1*) displayed low methylation levels and high expression. Six genes (*COL11A1*, *SDK2*, *BSX*, *CNTN5*, *ARID5B*, *KAZN*) showed high methylation levels with high expression. Four genes (*ATP2C2*, *PTPRT*, *CDYL2*, *DOK6*) demonstrated low methylation levels with low expression.
Cheng et al.^5^	Illumina Infinium MethylationEPIC, 850K BeadChip	68	Non‐invasive PitNETs	Non‐functioning	Invasiveness	Knosp grades 3–4	Increased methylation of *PHYHD1*, *LTBR*, *C22orf42*, *PRR5*, *ANKDD1A*, *RAB13*, *CAMKV*, *KIFC3*, *WNT4* and *STAT6* was observed in invasive tumours, as was decreased methylation of *MYBPHL*.
Cheng et al.^3^	Illumina Infinium MethylationEPIC, 850 K BeadChip	71	PitNETs without regrowth	Non‐functioning	Tumour Regrowth	The maximum tumour diameter has increased by more than 2 mm on enhanced MRI from surgery to follow‐up.	Increased DNA methylation levels were observed in *FAM90A1*, *ETS2*, *STAT6*, *CYBRD1* and *PYCARD* for tumour regrowth. Decreased methylation levels were observed in *MYT1L*, *ING2*, *KCNK1 and SH3GL2*. Patients who are younger, have decreased *FAM90A1* expression (HR = 0.233, 95% CI = 0.083–0.649) and increased *ING2* expression (HR = 3.020, 95% CI = 1.067–8.543) are more likely to experience tumour regrowth.
Garcia‐Martinez et al.	MS‐MLPA	105	Non‐invasive PitNETs	Non‐functioning, corticotroph and somatotroph	Invasiveness	Clinical manifestations, Hardy's classification, and MRI invasiveness of the cavernous sinus.	Compared to non‐invasive tumours, invasive tumours exhibited lower levels of methylation in *ESR1* and *RASSF1*.
Guaraldi et al.	Targeted bisulphite next‐generation sequencing (NGS)	111	Non‐Aggressive PitNETs	All subtypes, mainly non‐functioning	Aggressiveness	Tumour size >10 mm, extra‐sellar invasion (defined by Knosp grades 3–4/Hardy–Wilson stages D–E), high proliferation (ki‐67 ≥ 3% and p53 > 10% strongly positive nuclei/10 HPF or the presence of >2/10 mitoses/HPF), and requiring multiple treatment to obtain disease remission or, at least, control	The methylation levels of *PARP15*, *LINC00599* and *ZAP70* were higher in aggressive tumours. Conversely, the methylation levels of *AIP*, *GNAS* and *PDCD1* were lower in aggressive cases. Among X‐linked genes, males exhibited higher levels of methylation of *FLNA*, *UXT* and *MAGE* family genes (*MAGEA11*, *MAGEA1* and *MAGEC2*) in aggressive tumours.
Gu et al.	Illumina Infinium HumanMethylation, 450K Beadchip	19	Non‐invasive PitNETs	Non‐functioning	Invasiveness	Wilson–Hardy grades III–V and stages D–E	Cluster analysis of 339 CpGs revealed a clear distinction between invasive and non‐invasive tumours.
Hallen et al.	Illumina Infinium MethylationEPIC, 850K BeadChip	43	PitNETs without regrowth	Non‐functioning	Reintervention	Need for reoperation or radiotherapy due to tumour progression within 5 years of follow‐up	Hypermethylated DMPs in *GABRA1*, *ZNF664‐FAM101A* and *SLC23A1*, as well as hypomethylated DMPs in *CPED1* and *ATP2B4*, were associated with shorter reintervention‐free survival.
Jotanovic et al.	Illumina Infinium MethylationEPIC, 850K BeadChip	76	Non‐aggressive (benign) PitNETs	All subtypes, mainly corticotroph	Aggressiveness	Invasive tumour with unusually fast growth and/or clinically significant tumour progression despite surgery, radiotherapy, and standard medical therapy.	Unsupervised hierarchical clustering, based on the 5000 most variable CpG sites, revealed a clear separation between the aggressive tumour group and the benign tumour group. A total of 9066 significant DMPs were detected when aggressive and benign tumours were compared. Of these, 7394 DMPs exhibited hypermethylation and 1672 displayed hypomethylation.
Kayacan, et al.	MS‐PCR	32	Non‐invasive PitNETs	Corticotroph	Invasiveness	Knosp grades 3–4 or invasion of the bone and dura.	There was no difference in partial methylation status between invasive and non‐invasive tumours (50% vs. 47%, respectively).
Kober et al.	Illumina Infinium HumanMethylation, 450K Beadchip and DNA pyrosequencing assay.	34	Non‐invasive PitNETs	Non‐functioning	Invasiveness	Knosp grades 3–4 or invasion of the bone and dura of the sellar floor, sphenoid sinus or clivus and/or leptomeningeal infiltration.	No differences were observed in genome‐wide methylation analysis between invasive and non‐invasive tumours. However, *ITPKB* methylation was significantly lower in invasive tumours than in non‐invasive tumours (28% vs. 39%; *p* = .03), while *CNKSR1* methylation was higher in invasive tumours than in non‐invasive tumours (52% vs. 39%; *p* = .04).
Kochling et al.	MS‐PCR	100	Not applicable	All subtypes	Tumour recurrence and progression free survival	Tumour recurrence and progression free survival	*TERT* promoter methylation was observed in 27% of primary PitNETs and 33% of recurrent PitNETs. Furthermore, PFS was 44 and 95 months for methylated and unmethylated tumours, respectively.
Ling et al.	Illumina Infinium HumanMethylation, 450K Beadchip.	24	Non‐invasive PitNETs	All subtypes	Invasiveness	Knosp scores 2–4	No significant differences in global DNA methylation were observed between invasive and non‐ invasive PitNETs, nor was methylation associated with tumour grade. However, 34 CpG sites linked to 17 genes, including FLT1 and SLIT3, were found to be hypomethylated in invasive NF‐PitNETs.
Miyake et al.	MS‐HRM	70	Not applicable	All subtypes, but primarily non‐functioning	Progression free survival (PFS)	Time between the date of surgery until disease progression was confirmed by neuroimaging *Disease progression defined as*: (1) 30% increase in tumour volume (2) 10% increase in any dimension following incomplete resection (3) any detectable disease following complete resection.	Recurrent PitNETs exhibited a higher frequency of *TERT* promoter methylation (41%) than primary PitNETs (8%, *p* = .002). Furthermore, *TERT* promoter hypermethylation was associated with elevated *TERT* mRNA expression. The median PFS for methylated PitNETs was shorter (30 vs. 133 months), with *TERT* promoter methylation being associated with an increased risk of shorter PFS (HR: 5.804, 95% CI: 1.407–23.940).
Møller et al.	Illumina Infinium MethylationEPIC	42	Not applicable	Non‐functioning	Regrowth rate over time	Not applicable	No differences in tumour regrowth were observed between methylation clusters during the eight‐year follow‐up period.
Qian et al.	MS‐PCR	69	Non‐invasive PitNETs	All subtypes	Invasiveness	Wilson–Hardy grades III–V	Methylation of the *CDH13* gene was more prevalent in invasive PitNETs (42%) than in non‐invasive PitNETs (19%, *p* < .05). Furthermore, *CDH1* methylation was more prevalent in grade IV tumours than in grade I tumours.
Rusetska et al.	DNA pyrosequencing	80	Non‐invasive PitNETs	Non‐functioning	Invasiveness	Knosp grades 3–4 or invasion of the bone and dura of the sellar floor, sphenoid sinus or clivus and/or leptomeningeal infiltration.	Lower *LINE‐1* methylation levels were observed in invasive PitNETs (68%) than in non‐invasive ones (72%, *p* = .02).
Tsegaye, et al.	DNA pyrosequencing	47	Non‐invasive PitNETs	Non‐functioning	Invasiveness	Knosp grades 3–4 or invasion of the bone and dura of the sellar floor, sphenoid sinus or clivus and/or leptomeningeal infiltration.	No difference in hsa‐mir‐184 promoter methylation levels was observed between invasive and non‐invasive tumours. However, a higher level of hsa‐mir‐184 was observed in invasive tumours than in non‐invasive tumours.
Valiulyte et al.	MS‐PCR	88	Non‐invasive PitNETs	All subtypes, mainly lactotroph	Invasiveness	Wilson–Hardy grades III–V and suprasellar extension.	No difference in *STAT3* gene methylation was observed between invasive and non‐invasive tumours (12% vs. 7%, *p* = .43).
Yuan et al.	MS‐PCR	53	Non‐invasive PitNETs	All subtypes	Invasiveness	Wilson–Hardy grades III–V	*GSTP1* methylation was more prevalent in invasive tumours (85%) compared to non‐invasive tumours (63%, *p* < .05) and was associated with reduced gene expression.

Abbreviations: DMP, differentially methylated position; MS‐HRM, methylation‐sensitive high‐resolution melting analysis; MS‐MLPA, methylation‐specific multiplex ligation‐dependent probe amplification (MS‐MLPA); MS‐PCR, methylation‐specific polymerase chain reaction; NF‐PitNETs, non‐functioning pituitary neuroendocrine tumours; PFS, progression‐free survival; RRBS, reduced representation bisulphite sequencing.

**TABLE 2:** Overview of candidate genes for DNA methylation analysis for prediction of PitNETs behaviour.

Gene	Methylation status	Methylation assessment	Function	Gene expression	Related clinical outcome
*FAM90A1*	Increased	Illumina Infinium MethylationEPIC BeadChip	Member of the FAM90 family and a protein‐coding gene in human; specific function not well‐characterized	Decreased	Tumour regrowth
*EST2*	Increased	Illumina Infinium MethylationEPIC BeadChip	Transcription factor belonging to the ETS family, which regulates genes involved in development, apoptosis, and cellular proliferation.	Decreased	Tumour regrowth
*STAT6*	Increased	Illumina Infinium MethylationEPIC BeadChip	Transduces IL‐4 and IL‐13 signals; activates gene expression related to immune responses.	Decreased	Tumour regrowth, invasiveness
*CYBRD1*	Increased	Illumina Infinium MethylationEPIC BeadChip	Ferric reductase involved in dietary iron absorption; expressed in the duodenum.	Decreased	Tumour regrowth
*PYCARD* (*ASC*)	Increased	Illumina Infinium MethylationEPIC BeadChip	Mediator in apoptosis and inflammation; involved in inflammasome activation.	Decreased	Tumour regrowth
*MYT1L*	Decreased	Illumina Infinium MethylationEPIC BeadChip	Transcription factor that plays a critical role in neuronal development and maintenance of neuronal identity.	Increased	Tumour regrowth
*ING2*	Decreased	Illumina Infinium MethylationEPIC BeadChip	Tumour suppressor involved in DNA repair and apoptosis; modulates histone acetylation.	Increased	Tumour regrowth
*KCNK1*	Decreased	Illumina Infinium MethylationEPIC BeadChip	Potassium channel contributing to resting membrane potential in neurons.	Increased	Tumour regrowth
*KCK2*	Decreased	RRBS	Potassium channel gene involved in maintaining membrane potential.	Increased	Invasiveness
*SH3GL2*	Decreased	Illumina Infinium MethylationEPIC BeadChip	Involved in synaptic vesicle endocytosis and dendrite outgrowth; interacts with BDNF.	Increased	Tumour regrowth
*GABRA1*	Increased	Illumina Infinium MethylationEPIC BeadChip	Subunit of GABA‐A receptor; mediates inhibitory neurotransmission in the brain. Responsible for neuroactive ligand‐receptor interaction in the development of PitNETs.	Not studied	Re‐intervention free survival
*ZNF664‐FAM101A*	Increased	Illumina Infinium MethylationEPIC BeadChip	Dual role in transcriptional regulation and cellular processes. Involved in various diseases, including congenital diaphragmatic hernia, neurological disorders, and certain cancers.	Not studied	Re‐intervention free survival
*SLC23A1*	Increased	Illumina Infinium MethylationEPIC BeadChip	Key transporter involved in maintaining cellular vitamin C levels, which are crucial for various physiological processes.	Not studied	Re‐intervention free survival
*CPED1*	Decreased	Illumina Infinium MethylationEPIC BeadChip	Protein coding gene; function not fully characterized.	Not studied	Re‐intervention free survival
*ATP2B4*	Decreased	Illumina Infinium MethylationEPIC BeadChip	Encodes the plasma membrane calcium‐transporting ATPase 4 (PMCA4), a crucial enzyme responsible for maintaining low intracellular calcium levels by actively pumping calcium out of cells.	Not studied	Re‐intervention free survival
*PARP15*	Increased	Targeted bisulphite NGS	Member of the PARP family of proteins, which are involved in various cellular processes including DNA repair, chromatin remodelling, transcription, and cell death.	Not studied	Aggressiveness
*LINC00599* (*RNCR3*)	Increased	Targeted bisulphite NGS	Long non‐coding RNA that is involved in neural development, cardiovascular and pulmonary diseases and acts as a tumour suppressor.	Not studied	Aggressiveness
*ZAP70*	Increased	Targeted bisulphite NGS	Encodes the zeta‐chain‐associated protein kinase 70 (ZAP‐70), a crucial enzyme in the immune system's function, particularly in T cell development and activation.	Not studied	Aggressiveness
*AIP*	Decreased	Targeted bisulphite NGS	Tumour suppressor, that suppresses cell proliferation in PitNETs. Germline mutation mutations are associated with familial isolated pituitary tumours.	Not studied	Aggressiveness
*GNAS*	Decreased	Targeted bisulphite NGS	Encodes Gs alpha subunit; involved in signal transduction. Mutations associated with somatotroph PitNETs.	Not studied	Aggressiveness
*PDCD1*	Decreased	Targeted bisulphite NGS	Immune checkpoint receptor; regulates T‐cell activity	Not studied	Aggressiveness
*FLNA*	Increased	MS‐PCR	Cytoskeletal protein; links Actin filaments and participates in signalling pathways.	Not studied	Aggressiveness
*UXT*	Increased	MS‐PCR	Co‐chaperone modulating transcription; interacts with androgen receptor.	Not studied	Aggressiveness
*MAGE family*	Increased	MS‐PCR	Group of genes with roles in apoptosis and Tumour suppression; some members are cancer‐testis antigens.	Not studied	Aggressiveness
*TERT*	Increased	MS‐PCR	Regulates telomerase expression; mutations can lead to increased telomerase activity and cellular immortality.	Increased	Progression free survival
	Not significant	MS‐HRM		Not studied	Tumour recurrence
*PHYHD1*	Increased	Illumina Infinium MethylationEPIC BeadChip	Putative oxidoreductase; function not well‐characterized	Decreased	Invasiveness
*LTBR*	Increased	Illumina Infinium MethylationEPIC BeadChip	Receptor involved in lymphoid tissue development and apoptosis	Decreased	Invasiveness
*C22orf42*	Increased	Illumina Infinium MethylationEPIC BeadChip	Open reading frame; function not well‐characterized.	Decreased	Invasiveness
*PRR5*	Increased	Illumina Infinium MethylationEPIC BeadChip	Component of mTORC2 complex; involved in cell growth and survival.	Decreased	Invasiveness
*ANKDD1A*	Increased	Illumina Infinium MethylationEPIC BeadChip	Contains ankyrin repeats; function not well‐characterized.	Decreased	Invasiveness
*RAB13*	Increased	Illumina Infinium MethylationEPIC BeadChip	Member of RAS oncogene family; involved in vesicle trafficking.	Decreased	Invasiveness
*CAMKV*	Increased	Illumina Infinium MethylationEPIC BeadChip	Kinase‐like protein expressed in the brain; function not well‐characterized.	Decreased	Invasiveness
*KIFC3*	Increased	Illumina Infinium MethylationEPIC BeadChip	Motor protein involved in intracellular transport.	Decreased	Invasiveness
*WNT4*	Increased	Illumina Infinium MethylationEPIC BeadChip	Developmental gene crucial for female reproductive tract development, kidney morphogenesis, tissue regeneration, maintaining cellular homeostasis	Decreased	Invasiveness
*MYBPHL*	Decreased	Illumina Infinium MethylationEPIC BeadChip	Myosin‐binding protein; function not well‐characterized.	Increased	Invasiveness
*LINE‐1*	Decreased	DNA pyrosequencing	Autonomous retrotransposons that can copy and insert themselves into new locations in the genome and contributing to genetic variation, gene regulation, and genome evolution.	Increased	Invasiveness
*ITPKB*	Increased	DNA pyrosequencing	Kinase involved in inositol phosphate metabolism.	Decreased	Invasiveness
*CNKSR1*	Increased	DNA pyrosequencing	Scaffold protein, facilitating interactions between various signalling molecules, including those in the RAS/MAPK and PI3K/AKT pathways, which are vital for cell proliferation, survival, and differentiation.	Decreased	Invasiveness
*CDH13*	Increased	MS‐PCR	Cadherin involved in cell adhesion; acts as a tumour suppressor.	Decreased	Invasiveness
*CDH1*	Increased	MS‐PCR	E‐cadherin; critical for cell–cell adhesion; loss associated with tumour progression.	Decreased	Invasiveness
*GSTP1*	Increased	MS‐PCR	Enzyme that helps detoxify harmful compounds, protects cells from oxidative stress, and regulates cell signalling.	Decreased	Invasiveness
*ESR1*	Decreased	MS‐MLPA	Encodes the estrogen receptor alpha (ERα), a nuclear hormone receptor that regulates gene expression in response to estrogen.	Not studied	Invasiveness
*RASSF1*	Decreased	MS‐MLPA	Tumour suppressor involved in cell cycle regulation, apoptosis, and microtubule stability.	Not studied	Invasiveness
*STAT3*	Not significant	MS‐PCR	Transcription factor involved in cell growth and apoptosis.	Not significant	Invasiveness
*FGFR3*	Decreased	RRBS	Encodes a fibroblast growth factor receptor; mutations or overexpression can drive cell proliferation and survival, contributing to bladder, lung, and other cancers.	Increased	Invasiveness
*SSC5D*	Decreased	RRBS	Encodes a protein implicated in the development of neuroblastomas and other cancers; may affect cell adhesion or migration.	Increased	Invasiveness
*FBXO2*	Decreased	RRBS	Part of the ubiquitin‐proteasome pathway, regulating protein degradation; overexpression can contribute to tumour progression by modulating cell cycle and apoptosis.	Increased	Invasiveness
*SREBF1*	Decreased	RRBS	A transcription factor regulating lipid metabolism; overexpression can enhance lipid biosynthesis and support rapid tumour growth.	Increased	Invasiveness
*SLITRK1*	Decreased	RRBS	Involved in neural development and cell signalling; mutations or dysregulation may influence cancers, particularly neuroblastomas, by affecting neuronal growth.	Increased	Invasiveness
*COL11A1*	Increased	RRBS	Encodes a collagen type XI alpha chain; can promote tumour cell invasion and metastasis.	Increased	Invasiveness
*SDK2*	Increased	RRBS	A member of the immunoglobulin superfamily, involved in cell adhesion; altered expression in cancers could impact tumour progression and metastasis.	Increased	Invasiveness
*BSX*	Increased	RRBS	Involved in the regulation of the central nervous system, with potential roles in cancer by modulating cell differentiation or apoptosis.	Increased	Invasiveness
*CNTN5*	Increased	RRBS	A cell adhesion molecule; dysregulated expression is implicated in the development of neuroectodermal tumours like neuroblastomas.	Increased	Invasiveness
*ARID5B*	Increased	RRBS	A member of the ARID family, which regulates gene transcription; mutations can lead to disrupted cellular differentiation and oncogenesis.	Increased	Invasiveness
*KAZN*	Increased	RRBS	Associated with the regulation of the cytoskeleton and adhesion molecules, influencing tumour cell migration and invasion in cancers.	Increased	Invasiveness
*ATP2C2*	Decreased	RRBS	Encodes a calcium ATPase; involved in calcium signalling, which is important in cell proliferation, migration, and apoptosis in various cancers.	Decreased	Invasiveness
*PTPRT*	Decreased	RRBS	A receptor‐type protein tyrosine phosphatase; acts as a tumour suppressor by regulating cell signalling pathways involved in growth, differentiation, and migration.	Decreased	Invasiveness
*CDYL2*	Decreased	RRBS	Involved in chromatin remodelling and gene regulation; mutations or downregulation may contribute to the progression of haematological malignancies.	Decreased	Invasiveness
*DOK6*	Decreased	RRBS	Part of the docking protein family; interacts with receptor tyrosine kinases and may influence cell signalling pathways that regulate proliferation and survival.	Decreased	Invasiveness
*CDKN2A*	Not significant	MS‐PCR	Tumour suppressor encoding p16INK4a and p14ARF; crucial for cell cycle regulation; loss or inactivation leads to uncontrolled cell proliferation in many cancers.	Not Significant	Invasiveness
*Has‐miR‐184*	Not significant	MS‐PCR	MicroRNA involved in the regulation of tumour suppressor genes and oncogenes; its dysregulation can contribute to tumour progression in various cancers.	Increased	Invasiveness

*Note*: Information about gene functions retrieved from *Gene [Internet]*. Bethesda (MD): National Center for Biotechnology Information (US), National Library of Medicine; c2004 [cited 2025, October 10]. Available from: https://www.ncbi.nlm.nih.gov/gene/.

Abbreviations: MS‐HRM, methylation‐sensitive high‐resolution melting analysis; MS‐MLPA, methylation‐specific multiplex ligation‐dependent probe amplification (MS‐MLPA); MS‐PCR, methylation‐specific polymerase chain reaction; RRBS, reduced representation bisulphite sequencing.

#### 3.2.1 Genome‐wide DNA methylation analysis and PitNET invasiveness

Cheng et al.^5^ examined a cohort of 68 NF‐PitNETs with Illumina Infinium MethylationEPIC BeadChip array and found that invasive subtypes exhibited hypermethylation and decreased expression of genes such as *PHYHD1*, *LTBR*, *C22orf42*, *PRR5*, *ANKDD1A*, *RAB13*, *CAMKV*, *KIFC3*, *WNT4*, and *STAT6*. Conversely, *MYBPHL* exhibited hypomethylation and was overexpressed in invasive tumours.^5^

Notably, *LTBR*, a member of the TNF receptor superfamily involved in immune and inflammatory responses, showed downregulation in invasive tumours, suggesting a potential tumour‐suppressor role, though further research is needed to clarify its function.^44^ Similarly, *WNT4*, which encodes glycoproteins involved in cell proliferation, migration, and survival, may act as either a tumour suppressor or a pro‐oncogene, depending on the tumour context.^45^ In invasive PitNETs, hypermethylation and decreased expression of *WNT4* suggest it may function as a tumour suppressor in these tumours.

Another study by Gu et al.^7^ analysed 339 differentially methylated CpG sites (overall, methylation difference: |Δ*β*| > 0.1 and *p* < .001) in 19 NF‐PitNETs using the Illumina Infinium MethylationEPIC BeadChip, clearly distinguishing invasive from non‐invasive tumours. Gene Ontology (GO) analysis revealed that genes like *CARD11*, *GALNT9*, *FBXW8*, and *SEPT9* were associated with cell adhesion pathways, indicating a possible link to invasiveness. However, the methylation of *GALNT9* did not correlate with its gene expression levels.

The most recent genome‐wide study by Chen et al.^4^ expanded on this evidence using RRBS in a cohort of 41 PitNETs of multiple subtypes. The study identified 347 differentially methylated regions (DMRs) (|Δ*β*| > 0.1), of which 63% were hypomethylated in invasive tumours. Some of the genes associated with these DMRs displayed distinct methylation–expression patterns. For instance, *FGFR3*, *KCNK2*, *C4orf50*, *SSC5D*, *FBXO2*, *SREBF1*, and *SLITRK1* showed low methylation and high expression in invasive tumours, while *COL11A1*, *SDK2*, *BSX*, *CNTN5*, *ARID5B*, and *KAZN* demonstrated high methylation but increased expression. This suggests complex regulatory mechanisms beyond simple promoter silencing. Additionally, genes such as *ATP2C2*, *PTPRT*, *CDYL2*, and *DOK6* exhibited both low methylation and low expression, implying the involvement of non‐methylation‐dependent regulatory pathways. Overall, the combined methylation and mRNA expression profiles of these 17 genes effectively distinguished invasive from non‐invasive PitNETs.

Among these genes, *FGFR3* (fibroblast growth factor receptor 3) expression alterations have been implicated in cell proliferation and invasiveness in various cancers.^46^ Increased expression of *FGFR3* could affect fibroblast growth factor signalling, which could stimulate tumour progression and invasiveness in PitNETs.^4^ This was also seen for *SREBF1*, a transcription factor that regulates lipid biosynthesis, which is crucial for membrane formation and cell growth. Upregulation of *SREBF1* is associated with increased invasiveness in several cancers and may contribute to PitNET invasiveness.^47^ Increased expression of *COL11A1*, a component of type XI collagen involved in extracellular matrix (ECM) remodelling, has been linked to tumour invasion and metastases in multiple malignancies. This gene may also impact tumour cell adhesion, migration, and invasiveness in PitNETs.^48^ *PTPRT* encodes the protein tyrosine phosphatase and functions as a tumour suppressor, regulating cell signalling pathways related to growth, differentiation, and migration. It is frequently silenced by methylation in various cancers.^49^ Chen et al.^4^ demonstrated a strong correlation (*r* = 0.81) between decreased methylation and increased expression of *PTPRT* in invasive PitNETs, highlighting it as a promising biomarker for PitNET invasiveness.

Despite these new insights, two case control studies^12^, ^14^ that investigated genome‐wide DNA methylation analyses using Illumina Infinium HumanMethylation 450K Beadchip array found no significant methylation differences between invasive and non‐invasive tumours. Ling et al^14^ included multiple PitNET subtypes, and Kober et al.^12^ focused on NF‐PitNETs with Knosp scores ranging from 2 to 4 to define tumour invasion. Both studies were limited by small sample sizes (12 and 18 invasive cases, respectively), which may have limited their statistical power.

#### 3.2.2 Targeted gene methylation analysis and PitNET invasiveness

Rusetska et al.^18^ used DNA‐pyrosequencing to show that invasive tumours had significantly lower *LINE‐1* element methylation compared to non‐invasive tumours (mean 68.0% vs. 72%; *p* = .019, respectively). Although no significant differences were observed in transcript levels of L1‐ORF1 and L1‐ORF2, a negative correlation was found between *LINE‐1* methylation and transcript expression. LINE‐1 elements, which account for about 17%–20% of the human genome, are often used as a surrogate marker for global DNA methylation. Hypomethylation of *LINE‐1* is associated with genomic instability in various cancers. In this context, *LINE‐1* hypomethylation may contribute to aggressive behaviour in PitNETs.^50^

In the study of Kober et al.,^12^ that also applied DNA‐pyrosequencing, methylation of *ITPKB* was lower in invasive NF‐PitNETs (28%) compared to non‐invasive tumours (39%, *p* = .031). In contrast, *CNKSR1* methylation was higher in invasive tumours (52% vs. 39%, *p* = .043). Expression analysis revealed a strong inverse correlation between methylation and gene expression for both genes, with *CNKSR1* expression significantly reduced (fold change = 1.6) in invasive tumours.^12^ *CNKSR1* is a scaffold protein involved in critical signalling pathways, such as RAS/MAPK and PI3K/AKT, which regulate proliferation and survival, suggesting its potential involvement in PitNET invasiveness.^51^

Qian et al.,^17^ using MS‐PCR, reported that promoter methylation of *CDH13* was more frequent in invasive tumours compared to non‐invasive ones (42% vs. 19%; *p* < .05). Additionally, CDH1 methylation levels were higher in Wilson‐Hardy grade IV tumours than in grade I tumours. Both *CDH1* and *CDH13* showed decreased expression in invasive tumours.^17^ *CDH1* encodes E‐cadherin, a key adhesion molecule whose loss is a hallmark of epithelial‐to‐mesenchymal transition (EMT), associated with tumour invasion.^52^ *CDH13* encodes H‐cadherin, which plays a role in cell migration and proliferation, and its downregulation is also linked to increased invasiveness.^53^

Yuan et al.,^21^ also using MS‐PCR, demonstrated that GSTP1 promoter methylation was significantly higher in invasive tumours (85%) compared to non‐invasive tumours (63%; *p* < .05), with a corresponding decrease in expression. As *GSTP1* protects against oxidative damage, its silencing through hypermethylation has been linked to increased DNA damage and tumour progression in several cancers, potentially contributing to invasive PitNET behaviour.^54^

In contrast, García‐Martínez et al.^6^ found that the estrogen receptor *ESR1* and the tumour suppressor gene *RASSF1* were less methylated in invasive tumours compared to non‐invasive ones (*p* = .05 and *p* = .03, respectively), although their expression profiles were not assessed in this study.

Valiulyte et al.^20^ found no significant difference in the methylation of *STAT3*, a transcription factor involved in cell growth and apoptosis, between invasive and non‐invasive tumours (12% vs. 7%; *p* = .43).

A more recent study by Tsegaye et al.^19^ analysed hsa‐miR‐184 promoter methylation in 47 gonadotroph PitNETs using pyrosequencing. Although no significant difference was found between invasive and non‐invasive tumours, hsa‐miR‐184 expression was significantly higher in invasive tumours (*p* = .03). This suggests that while methylation of hsa‐miR‐184 may not directly correlate with invasiveness, post‐transcriptional regulation involving this microRNA could play a role in the invasive behaviour of tumours. miR‐184 has been reported as a tumour suppressor in various cancers by inhibiting proliferation and invasion. Its overexpression may promote cell proliferation and inhibit apoptosis, potentially contributing to the invasive behaviour of gonadotroph PitNETs.^55^

Lastly, Kayacan et al.^11^ investigated *CDKN2A* promoter methylation in 32 corticotroph PitNETs using MS‐PCR and found no significant differences in methylation between invasive and non‐invasive tumours (partial methylation: 50% vs. 47%). Furthermore, *CDKN2A* expression did not correlate with tumour invasiveness.

### 3.3 PitNET aggressiveness

The relationship between DNA methylation patterns and aggressive behaviour in PitNETs has been investigated in two case–control studies.^8^, ^10^ Both studies used high‐throughput methylation techniques, including targeted bisulphite next‐generation sequencing (NGS) and Illumina Infinium MethylationEPIC (850 K) BeadChip arrays. These analyses covered a variety of PitNET subtypes, including corticotroph, somatotroph, gonadotroph, lactotroph, thyrotroph, plurihormonal Pit1+, and null cell tumours. Detailed study characteristics and gene‐level findings are summarized in Tables 1 and 2.

Guaraldi et al.^8^ identified distinct methylation profiles in 111 PitNETs using NGS, comparing aggressive (including 6 pituitary carcinomas) and non‐aggressive tumours. Aggressive PitNETs and carcinomas were defined by their size (>10 mm), extra‐sellar invasion (based on Knosp grades 3–4 or Hardy–Wilson stages D–E), high proliferation (Ki‐67 ≥ 3% and p53 > 10% strongly positive nuclei per 10 HPF), or metastatic spread to the central nervous system or distant sites for carcinomas. They found that aggressive tumours exhibited higher methylation of *PARP15*, *LINC00599*, and *ZAP70*, and lower methylation of *AIP*, *GNAS*, and *PDCD1* (*p* < .05 for all comparisons). In males, increased methylation of several X‐linked genes (*FLNA*, *UXT*, *MAGEA11*, *MAGEA1*, and *MAGEC2*) was also associated with tumour aggressiveness.

*PARP15* has been linked to tumour‐infiltrating lymphocytes and overall survival in lung adenocarcinoma,^56^ with higher methylation potentially reducing its expression thereby contributing to immune evasion and tumour aggressiveness. Similarly, *LINC00599* (*RNCR3*), a tumour suppressor gene downregulated in gliomas, may promote tumour growth when hypomethylated.^57^ Although the expression profiles of these genes in PitNETs with altered methylation remain unexamined, they may play a role in PitNET aggressiveness.

*AIP*, frequently mutated in familial isolated pituitary adenomas, is associated with early‐onset, treatment‐resistant tumours.^58^, ^59^ *GNAS*, which encodes Gsα, is frequently mutated in somatotroph PitNETs. However, somatotroph PitNETs with *GNAS* mutations are typically small and less invasive.^60^ Hypomethylation of these genes may contribute to more aggressive tumour behaviour, as suggested by Guaraldi et al.^8^ *PDCD1* encodes PD‐1, a checkpoint receptor that suppresses immune responses when bound by PD‐L1. Hypomethylation of PDCD1 may lead to overexpression of PD‐1, facilitating immune evasion and tumour progression.^61^ Expression profiling of these genes in PitNETs with altered methylation status has not yet been conducted.

In parallel, Jotanović et al.^10^ performed a genome‐wide methylation analysis using the Illumina Infinium MethylationEPIC (850 K) BeadChip in 76 patients, including 48 aggressive adenomas, 16 pituitary carcinomas, and 23 non‐aggressive PitNETs. Aggressive PitNETs and carcinomas were defined by invasive growth, rapid progression, or clinically significant progression despite surgery, radiotherapy, or standard medical therapy, with carcinomas characterized by metastatic spread to the central nervous system or distant sites. Unsupervised hierarchical clustering based on the 5000 most variable CpG sites achieved complete separation between the aggressive/pituitary carcinoma (APT/PC) group and benign tumours, indicating distinct global methylation signatures. The analysis identified 9066 significantly differentially methylated positions (DMPs) (|Δ*β*| ≥ 0.2, *p* < 1.3 × 10^−7^), including 7394 hypermethylated and 1672 hypomethylated CpG sites in aggressive tumours and carcinomas compared to benign PitNETs. These results underscore the extent to which epigenetic reprogramming occurs in clinically aggressive and malignant PitNETs, suggesting that widespread DNA methylation alterations may drive the transition from indolent to aggressive or metastatic phenotypes.

### 3.4 PitNET regrowth, recurrence and re‐intervention

The relationship between DNA methylation patterns and regrowth, recurrence and re‐intervention in PitNETs was examined in five studies with sample sizes ranging from 42 to 100. Two of these were case–control studies^3^, ^9^ and one retrospective cohort study^16^ focused on tumour regrowth and reintervention in NF‐PitNETs using genome‐wide DNA methylation profiling. The other two retrospective studies^13^, ^15^ examined targeted gene methylation using MS‐PCR and Methylation‐Sensitive High‐Resolution Melting (MS‐HRM) across various PitNET subtypes, including lactotroph, corticotroph, somatotroph, gonadotroph and thyrotroph, as well as plurihormonal tumours and null cell tumours. Detailed study characteristics and gene‐level findings are summarized in Tables 1 and 2.

#### 3.4.1 Genome‐wide DNA methylation analysis

Cheng et al.^3^ investigated the association between genome‐wide DNA methylation and PitNET regrowth defined by more than 2 mm of the maximum tumour diameter on enhanced MRI from the day of surgery to the follow‐up endpoint in 71 NF‐PitNETs. The study identified significant epigenetic differences between tumours with and without regrowth. In particular, hypermethylation and downregulation were observed in genes like *FAM90A1*, *ETS2*, *STAT6*, *CYBRD1*, and *PYCARD*. Meanwhile *MYT1L*, *ING2*, *KCNK1*, and *SH3GL2* were hypomethylated and upregulated in tumours with regrowth. In multivariable Cox regression, younger age (HR = 0.32, *p* = .02), decreased *FAM90A1* expression (HR = 0.23, *p* = .005), and increased *ING2* expression (HR = 3.02, *p* = .04) emerged as independent predictors of tumour regrowth.^3^ Although *ETS2* and *MYT1L* were included in the model, variables such as sex, tumour volume, invasion status, and expression of *STAT6* or *KCNK1* were excluded due to lack of significance in univariable analyses.

Several of these genes have previously been linked to tumour progression, which can contribute to tumour regrowth in PitNETs. For example, *ETS2*, a proto‐oncogene involved in development, apoptosis, and cell proliferation, is usually overexpressed in various cancers.^62^ However, *ETS2* was downregulated in NF‐PitNETs with regrowth compared to those with no regrowth. This unexpected finding may suggest that *ETS2* plays a tumour‐suppressive role that is unique to pituitary tumours.

*PYCARD* was found to be hypermethylated and down‐regulated in NF‐PitNETs with regrowth relative to the ones without regrowth.^3^ It plays a complex and somewhat contradictory role in neoplasms, as it can both promote inflammation and tumour growth, but also act as a tumour suppressor by inducing apoptosis and inhibiting tumour cell proliferation. In this context, the downregulation of *PYCARD* in NF‐PitNETs with regrowth suggests that it would primarily act as a tumour suppressor in these tumours.^63^

*ING2*, known for its tumour‐suppressive properties, was found to have increased expression in recurrent PitNETs. This is contrary to the typical expectation that ING2 loss promotes tumour progression.^64^ This unexpected upregulation suggests the involvement of context‐dependent regulation or compensatory mechanisms in PitNETs.

*KCNK1*, which encodes the TWIK‐1 potassium channel, has been associated with tumour cell migration and poor prognosis in several cancers. Overexpression of *KCNK1* in NF‐PitNETs may contribute to increased tumour invasiveness and aggressiveness.^65^, ^66^ *SH3GL2*, involved in intracellular trafficking and cell signalling, is usually downregulated in cancers and linked to increased invasiveness via the STAT3/MMP2 pathway. Loss of *SH3GL2* expression in PitNETs may promote tumour regrowth.^67^ Finally, *FAM90A1*, identified as an independent prognostic marker, shows decreased expression associated with tumour regrowth^3^ though its exact biological function remains unclear and warrants further research.

In another study by Møller et al.,^16^ methylation‐based clustering analysis was used to predict tumour regrowth in 42 large PitNETs. However, due to small cluster sizes (ranging from 4 to 10 participants), the study was unable to reliably predict regrowth outcomes over a median follow‐up of 75 months. While one of the five clusters showed increased cumulative regrowth over time in a linear mixed‐effects model, this pattern did not differ from other methylation‐based clusters, likely due to limited statistical power.^16^

When examining reintervention‐free survival, Hallen et al^9^ found that in 43 non‐functioning PitNETs shorter survival was significantly associated with hypermethylation of DMPs in *GABRA1*, *ZNF664‐FAM101A*, and *SLC23A1*.^9^ In contrast, hypomethylation was observed in *CPED1* and *ATP2B4*, although gene expression profiles were not assessed in this study.

*GABRA1* encodes the α1 subunit of the GABA receptor and is involved in central nervous system signalling. Its specific role in human PitNETs remains unclear, but it may act as a central regulatory hub in tumour progression.^68^ Similarly, *ATP2B4*, which regulates intracellular calcium homeostasis, has been associated with increased cell migration and decreased apoptosis in other malignancies. Hypomethylation of *ATP2B4* in PitNETs suggests potential upregulation, which contributes to aggressive tumour behaviour and shorter reintervention‐free survival.^69^

#### 3.4.2 Targeted gene methylation analysis

Köchling et al.^13^ investigated *TERT* promoter methylation status in 85 primary and 15 recurrent PitNETs across all subtypes using MS‐PCR. Their study found no association between *TERT* promoter methylation status and tumour recurrence or progression‐free survival.^13^ However, the study by Miyake et al.^15^ using MS‐HRM in 70 PitNETs (mainly non‐functioning (*n* = 59)) showed that *TERT* promoter methylation was significantly more frequent in recurrent tumours (41%) than in primary tumours (8%) (*p* = .003). Furthermore, *TERT* methylation was associated with higher expression and shorter progression‐free survival (hazard ratio [HR] = 5.80, 95% confidence interval [CI]: 1.41–23.94; *p* = .02), even after adjusting for age, sex, tumour size, extent of resection, and the presence of a primary or recurrent tumour.^15^ These findings align with the known paradoxical relationship between *TERT* promoter hypermethylation and TERT increased expression in promoting telomerase activity, cellular immortality, and tumour recurrence.^70^, ^71^, ^72^, ^73^, ^74^

### 3.5 Clinical implications for prediction modelling

To gain a structured and comprehensive understanding of the clinical and DNA‐methylation‐related predictive factors of PitNET behaviour, we reviewed existing studies and assessed their potential clinical implications for predictive modelling (Table 3). Several demographic factors (age, sex), tumour characteristics (size, subtype, Ki‐67 index, p53 overexpression), and disease‐related factors (hormone hypersecretion, post‐operative hormone levels) have been identified as predictors of several tumour behaviour outcomes.^2^, ^22^, ^75^, ^76^, ^77^, ^78^, ^79^

**TABLE 3:** Overview of predictive factors for outcomes concerning PitNET behaviour.

Outcomes	Consensus definitions	Demographic	Tumour‐related	Surgical	Disease‐related	DNA Methylation alternation of Gene(s)
Tumour invasiveness	Knosp grade 3 or 4 and/or Hardy–Wilson stage D or E	Age, sex	Tumour size, tumour subtype		Hormone hypersecretion	*PHYHD1*, *LTBR*, *C22orf42*, *PRR5*, *ANKDD1A*, *RAB13*, *CAMKV*, *KIFC3*, *WNT4*, **STAT6**, *MYBPHL*, *FGFR3*, *KCNK2*, *C4orf50*, *SSC5D*, *FBXO2*, *SREBF1*, *SLITRK1*, *COL11A1*, *SDK2*, *BSX*, *CNTN5*, *ARID5B*, *KAZN*, *ATP2C2*, *PTPRT*, *CDYL2*, *DOK6*, *LINE‐1*, *ITPKB*, *CNKSR1*, *CDH1*, *CDH13*, *GSTP1*, *ESR1 and RASSF1*
Tumour aggressiveness	Tumour size >10 mm, extra‐sellar invasion (defined by Knosp grades 3–4/Hardy–Wilson stages D–E), high proliferation (ki‐67 ≥ 3% and p53 > 10% strongly positive nuclei/10 HPF or the presence of >2/10 mitoses/HPF), and requiring multiple treatment to obtain disease remission or, at least, control.	Age, sex	Tumour size, tumour subtype, ki‐67 index, p53 overexpression, cavernous sinus	Extend of resection, surgical complications.	Hormone hypersecretion, post‐operative hormone levels.	*PARP15*, *LINC005*99, *ZAP70*, *AIP*, *GNAS*, *PDCD1*, *FLNA*, *FLNA*, *UXT*, *MAGE family*
Progression‐free survival	Time from the date of surgery until disease progression defines as: (1) 30% increase in tumour volume (2) 10% increase in any dimension following incomplete resection (3) any detectable disease following complete resection.	Age, sex	Tumour size, tumour subtype, ki‐67 index, p53 overexpression, cavernous sinus	Extend of resection, Surgical complications.	Hormone hypersecretion, post‐operative hormone levels.	*TERT* ^a^
Tumour regrowth	Increase in maximum tumour diameter > 2 mm on enhanced MRI from surgery to follow‐up	Age, sex	Tumour size, tumour subtype, ki‐67 index, p53 overexpression, cavernous sinus invasion.	Extend of resection, surgical complications.	Hormone hypersecretion, post‐operative hormone levels.	*FAM90A1* ^a^, *ETS2*, **STAT6**, *CYBRD1*, *PYCARD*, *MYT1L*, *ING2* ^a^, *KCNK1*, and *SH3GL2*
Re‐intervention	Need for reoperation or radiotherapy due to tumour progression within 5 years of follow‐up	Age, sex	Tumour size, tumour subtype, ki‐67 index, p53 overexpression, cavernous sinus	Extend of resection, surgical complications.	Hormone hypersecretion, post‐operative hormone levels.	*GABRA1*, *ZNF664‐FAM101A*, *SLC23A1*, *CPED1*, *ATP2B4*

^a^ Assessed in a multivariable prediction model.

Tumour invasiveness (e.g., Knosp grade 3–4 and/or Hardy–Wilson stage D–E) was associated with changes in mean DNA methylation levels in genes such as *PHYHD1*, *STAT6*, *RASSF1*, *ESR1*, *SREBF1*, *COL11A1*, *PTPRT*, *LINE‐1*, *CDH1*, *CDH13*, and *STAT3*. Aggressive tumour behaviour, characterised by larger size, extrasellar invasion, high proliferation, and the requirement for multiple treatments, was associated with alterations in mean DNA methylation levels in the genes *PARP15*, *ZAP70*, *GNAS*, *AIP*, and *FLNA*.

TERT methylation emerged as a key predictive marker for tumour recurrence and reduced progression‐free survival. MRI‐assessed tumour regrowth was associated with changes in mean DNA methylation levels in *FAM90A1*, *STAT6*, *ING2*, and *SH3GL2*, while the need for re‐intervention correlated with changes in methylation levels in *GABRA1*, *ZNF664‐FAM101A*, *SLC23A1*, and *ATP2B4*. Notably, hypermethylation of the *STAT6* gene was found to be a potential predictor of both invasive behaviour and regrowth after surgery (see Table 3, highlighted in bold).^3^, ^5^ These findings emphasise the importance of combining epigenetic and clinical markers to improve prognostic accuracy and guide personalized treatment strategies for PitNETs.

## 4 DISCUSSION

This systematic review provides a comprehensive evaluation of DNA methylation as a predictive marker for PitNET behaviour. The findings highlight the growing interest in utilizing epigenetic biomarkers to improve prognostication, particularly in NF‐PitNETs, which present significant clinical challenges due to their typically asymptomatic hormonal profiles.

Our review identifies several promising candidate genes that could serve as methylation‐based biomarkers. These genes, including *PHYHD1*, *LTBR WNT4*, *STAT6*, *MYBPHL*, *FGFR3*, *SREBF1*, *COL11A1*, *PTPRT*, *LINE‐1*, *ITPKB*, *CNKSR1*, *CDH1*, *CDH13*, *GSTP1*, *ESR1*, *RASSF1*, and *STAT3*, were found to be differentially methylated in tumours exhibiting invasive behaviour. Additionally, *PARP15*, *AIP*, *GNAS* and *PDCD1*, were linked to aggressive tumour behaviour. *TERT*, *FAM90A1*, *ETS2*, *STAT6*, *CYBRD1*, *PYCARD*, *ING2*, *CPED1*, and *ATP2B4* were associated with tumour regrowth, recurrence, and the need for reintervention.

These genes are known to play oncogenic or tumour‐suppressive roles in various cancer types, and their upregulation or downregulation may contribute to the aggressive or invasive behaviour of PitNETs. This suggests that their methylation status could serve as valuable predictors of clinical outcomes.

However, several challenges complicate the interpretation of these findings. One major issue is the variability in reported outcomes related to tumour behaviour. Some studies assessed tumour invasiveness using radiological or histopathological criteria, while others focused on clinical endpoints such as tumour regrowth, reoperation, or radiotherapy. This inconsistency in outcome definitions limits the comparability of results across studies, highlighting the need for standardized clinical outcome measures when assessing the clinical predictive value of DNA methylation analysis in future research.

Additionally, many studies had small sizes, were single‐centre, and retrospective, introducing potential selection bias and confounding factors. In terms of the reproducibility and validation of genes with altered DNA methylation, only one gene, STAT6, was found to be hypermethylated in two separate studies that performed genome‐wide DNA methylation analysis in invasive pituitary tumours and tumours that showed regrowth after surgery.^3^, ^5^ However, none of the studied genes were validated in a subsequent study with the same tumour behaviour outcome. Notably, only a limited number of studies used multivariable predictive modelling, which is essential for evaluating the individual predictive effect of methylation markers in addition to the established clinical predictors.

Another limitation is the inconsistent classification of PitNET subtypes and tumour sizes. Many studies predate the 2022 WHO classification system, which is based on transcription factor profiling.^75^ As a result, patient cohorts were often heterogeneous. While most studies focused on non‐functioning PitNETs, some included all PitNET subtypes, which further limits comparability. This is important, as lineage‐driven methylation patterns underlie major differences among PitNET subtypes,^37^, ^38^ especially in studies which included multiple PitNET subtypes.^4^, ^8^, ^10^, ^13^, ^14^, ^15^, ^17^, ^20^, ^21^ Some studies took this into account, analysing the relationship between DNA methylation and PitNET subtype and performing stratified analyses or correcting for subtype‐specific methylation.^13^, ^15^, ^20^, ^21^ While few studies found that PitNET subtypes were similarly distributed between invasive and non‐invasive tumours^8^, ^14^ or between aggressive and non‐aggressive subtypes,^8^ others observed differences in the distribution of subtypes.^4^, ^10^ This inconsistency in adjusting for PitNET subtypes can introduce bias when interpreting the associations between DNA methylation and tumour behaviour. Therefore, caution is necessary when drawing conclusions from these findings.

Despite these limitations, our review has several strengths. This is the first systematic review in this field to incorporate a formal quality assessment of the included studies using the QUIPS tool,^42^ ensuring that only studies with sufficient validity were considered in assessing the predictive potential of methylation markers for clinical use.

Besides, we provide a comprehensive overview of candidate genes, detailing their methylation status, expression profiles, and functional roles, as well as the methodologies used for methylation analysis. Furthermore, we examine these genes in conjunction with established clinical and histological markers to assess their combined potential for predicting specific outcomes related to PitNET behaviour. These findings support the integration of epigenetic markers into future multivariable prediction models, which could be used in clinical practice. Incorporating these models could improve early detection of invasive and aggressive PitNETs, guide treatment selection, and enhance follow‐up strategies, ultimately leading to better patient outcomes and quality of life.

In conclusion, DNA methylation markers represent a promising tool for refining risk stratification in PitNETs, particularly in non‐functioning subtypes. However, their clinical implementation will require overcoming current methodological limitations. Future studies should adopt standardized outcome definitions, implement robust multivariable predictive models, and validate individual gene‐level methylation findings in larger, prospective cohorts. While global methylation patterns show promise, validation of individual gene‐level methylation markers as predictive biomarkers remains necessary. Current data remain inconsistent across studies and do not yet justify widespread clinical use.

## AUTHOR CONTRIBUTIONS

**Romy van der Groef:** Conceptualization; investigation; writing – original draft; formal analysis; validation; visualization; writing – review and editing; data curation; methodology. **Eskeatnaf Mulugeta:** Investigation; writing – original draft; writing – review and editing; validation; supervision. **Sebastian Neggers:** Writing – review and editing; conceptualization; validation; supervision. **Julie Refardt:** Conceptualization; investigation; writing – original draft; validation; methodology; visualization; writing – review and editing; supervision; data curation.

## FUNDING INFORMATION

No specific funding was received for this study.

## CONFLICT OF INTEREST STATEMENT

The authors declare no conflicts of interest.

## Supporting information


**APPENDIX S1:** Supporting information.

## Data Availability

The data that support the findings of this study are available from the corresponding author upon reasonable request.
