# Supplementary material for: DNA methylation as a predictor of pituitary neuroendocrine tumour behaviour: A systematic review
Source: J Neuroendocrinol. 2026 Mar 22;38(4):e70167. doi: 10.1111/jne.70167 (PMC13006151; doi:10.1111/jne.70167)
Supplement: Supplementary file 1 — APPENDIX S1: Supporting information. [file JNE-38-e70167-s001.docx]

**Supplementary Materials**

**S1. Search strategy**

| **Database searched** | **Platform** | **Years of coverage** | **Records** | **Records after duplicates removed** |
| --- | --- | --- | --- | --- |
| Medline ALL | Ovid | 1946 - 2025 | 371 | 368 |
| Embase | Embase.com | 1971 - 2025 | 752 | 414 |
| Web of Science Core Collection* | Web of Knowledge | 1975 - 2025 | 462 | 115 |
| Cochrane Central Register of Controlled Trials | Wiley | 1992 - 2025 | 0 | 0 |
| Additional Search Engines: Google Scholar** (100 top-ranked) | | | 100 | 49 |
| **Total** | | | **1685** | **946** |

*Science Citation Index Expanded (1975-2025) ; Social Sciences Citation Index (1975-2025) ; Arts & Humanities Citation Index (1975-2025) ; Conference Proceedings Citation Index- Science (1990-2025) ; Conference Proceedings Citation Index- Social Science & Humanities (1990-2025) ; Emerging Sources Citation Index (2005-2025)

**Google Scholar was searched via "Publish or Perish" to download the results in EndNote.

No other database limits were used than those specified in the search strategies

**Embase**

('hypophysis tumour'/exp OR 'pituitary neuroendocrine tumour'/de OR (((pituitary OR hypophys*) NEAR/3 (neoplas* OR tumour* OR tumour* OR malign* OR adenom* OR carcinom* OR incidentalom* OR macroadenom* OR microadenom*)) OR pitNET OR prolactinom* OR ((growth-hormone* OR GH) NEAR/6 (secret* OR produc*) NEAR/6 (adenom* OR tumour* OR tumour*))):ab,ti,kw) **AND** (epigenome/exp OR methylomics/de OR epigenetics/de OR 'DNA histone interaction'/de OR ' DNA methylation '/de OR ' histone modification'/exp OR (methylom* OR epigenom* OR epigenetic* OR ((DNA) NEAR/6 (histone*) NEAR/6 (modification* OR interact*)) OR methylat* OR hypermethyl* OR hypomethylat* OR ((histon*) NEAR/3 (modificat* OR acetylat* OR code* OR mark*))):ab,ti,kw) AND [ENGLISH]/lim NOT ([animals]/lim NOT [humans]/lim)

**Medline**

(exp Pituitary Neoplasms/ OR (((pituitary OR hypophys*) ADJ3 (neoplas* OR tumour* OR tumour* OR malign* OR adenom* OR carcinom* OR incidentalom* OR macroadenom* OR microadenom*)) OR pitNET OR prolactinom* OR ((growth-hormone* OR GH) ADJ6 (secret* OR produc*) ADJ6 (adenom* OR tumour* OR tumour*))).ab,ti,kf.) **AND** (Epigenome/ OR Epigenomics/ OR DNA Methylation/ OR Histone Code/ OR (methylom* OR epigenom* OR epigenetic* OR ((DNA) ADJ6 (histone*) ADJ6 (modification* OR interact*)) OR methylat* OR hypermethyl* OR hypomethylat* OR ((histon*) ADJ3 (modificat* OR acetylat* OR code* OR mark*))).ab,ti,kf.) AND english.la. NOT (exp Animals/ NOT Humans/)

**Cochrane**

((((pituitary OR hypophys*) NEAR/3 (neoplas* OR tumour* OR tumour* OR malign* OR adenom* OR carcinom* OR incidentalom* OR macroadenom* OR microadenom*)) OR pitNET OR prolactinom* OR ((growth-hormone* OR GH) NEAR/6 (secret* OR produc*) NEAR/6 (adenom* OR tumour* OR tumour*))):ab,ti,kw) **AND** ((methylom* OR epigenom* OR epigenetic* OR ((DNA) NEAR/6 (histone*) NEAR/6 (modification* OR interact*)) OR methylat* OR hypermethyl* OR hypomethylat* OR ((histon*) NEAR/3 (modificat* OR acetylat* OR code* OR mark*))):ab,ti,kw)

**Web of Science**

TS=(((((pituitary OR hypophys*) NEAR/2 (neoplas* OR tumour* OR tumour* OR malign* OR adenom* OR carcinom* OR incidentalom* OR macroadenom* OR microadenom*)) OR pitNET OR prolactinom* OR ((growth-hormone* OR GH) NEAR/5 (secret* OR produc*) NEAR/5 (adenom* OR tumour* OR tumour*)))) **AND** ((methylom* OR epigenom* OR epigenetic* OR ((DNA) NEAR/5 (histone*) NEAR/5 (modification* OR interact*)) OR methylat* OR hypermethyl* OR hypomethylat* OR ((histon*) NEAR/2 (modificat* OR acetylat* OR code* OR mark*)))) NOT ((animal* OR rat OR rats OR mouse OR mice OR murine OR dog OR dogs OR canine OR cat OR cats OR feline OR rabbit OR cow OR cows OR bovine OR rodent* OR sheep OR ovine OR pig OR swine OR porcine OR veterinar* OR chick* OR zebrafish* OR baboon* OR nonhuman* OR primate* OR cattle* OR goose OR geese OR duck OR macaque* OR avian* OR bird* OR fish*) NOT (human* OR patient* OR women OR woman OR men OR man))) AND LA=(English)

**Google Scholar**

"pituitary|hypophysis neoplasm|tumour|tumour|malignancy|adenoma|carcinoma|incidentaloma|macroadenoma|microadenoma|prolactinoma" methylome|methylomics|epigenome|epigenomics|epigenetics|methylation|hypermethylation|hypomethylation -animal -mouse -rat

'pituitary|hypophysis neoplasm|tumour|tumour|malignancy|adenoma|carcinoma|incidentaloma|macroadenoma|microadenoma|prolactinoma' methylome|methylomics|epigenome|epigenomics|epigenetics|methylation|hypermethylation|hypomethylation -animal -mouse -rat

**S2. Inclusion criteria**

**Inclusion criteria**

1. Randomised controlled trials (RCTs), non-randomised controlled trials (Non-RCTs), cohort studies, case-control studies and case series, providing evidence with regard to Pituitary Tumour behaviour AND DNA methylation/methylomics.
2. written in English

**Exclusion criteria**

1. Studies focusing only on Rathke cleft cysts and craniopharyngiomas.
2. Studies that focused on the methylation status of a single gene without an established biological function in tumour behaviour.
3. Studies that focused solely on tumour size.
4. Studies performing DNA methylation analyses only on cell-free DNA.
5. Animal-only studies
6. Studies including patients under 18 years of age
7. Reviews
8. Comments, abstracts, courses, seminars
9. Publications in languages other than English
10. No full-text available

**S3. Evidence tables**

|  | Study | Study design,  Surgery era,  Years of follow-up | Participants | Methylation analysis | Main outcomes | Additional remarks |
| --- | --- | --- | --- | --- | --- | --- |
| 1 | Chen, et al | Study design:  Case-control    surgery era:  2007-2016    Follow-up:  unknown | Diagnoses:  PitNETs:   - Corticotroph: 6 - Gonadotroph: 16 - Lactotroph: - Somatotroph: 2 - Null cell: 9 - Unknown: 4     Number of participants  41    Important exclusion criteria  Not reported    Age at surgery  Cases: mean 58.87 years, SD 12.14  Controls: 46.90, SD 12.33    Sex  22 males, 19 females    Cases  31 invasive PitNETs    Invasiveness:  histological/radiological evidence of tumour and/or surgical evidence of tumour invasion into the cavernous sinus or sphenoid sinus    Controls:  10 non-invasive PitNETs | Tissue sampling method  Immediately processed or frozen at -80 ◦C    Method  RRBS    Methylation analysis, n samples  41    Ki-analysis presence  No    Methylation analysis control  NA | Outcomes:  Differentially methylated regions  DMGs    Results:  347 regions with differential methylation (\|∆β\|> 0.1) between the groups. Notably, 63% (219 of 347) of these DMRs were hypomethylated.    Seven genes (FGFR3, KCNK2, C4orf50, SSC5D, FBXO2, SREBF1, SLITRK1) displayed low methylation levels and high expression, 6 genes (COL11A1, SDK2, BSX, CNTN5, ARID5B, KAZN) showed high methylation levels with high expression, and 4 genes (ATP2C2, PTPRT, CDYL2, DOK6) demonstrated low methylation levels with low expression.  The DNA methylation and mRNA levels of 17 genes effectively differentiate invasive from non-invasive PitNETs.  Notably, only protein tyrosine phosphatase receptor type T (PTPRT) exhibited a remarkably high correlation (r = 0.81) between its DNA methylation levels and mRNA expression levels. | Strengths:  - Combining DNA methylation and mRNA expression profile analyses    Limitations:  - Small sample size of the controls (n = 10).  - No multivariable predictive analysis    Risk of bias:  A. Selection bias:  Intermediate risk due to limited information about the selection procedure and inclusion and exclusion criteria.    B. Attrition bias:  Low risk due to complete follow-up.    C. Detection/information bias:  Intermediate risk. No mention of blinding the outcome assessor for invasiveness of the PitNETs or other patient factors. However, a standardized protocol for methylation analysis of PitNETs was performed.    D. Confounding:  Intermediate: the baseline table indicates a relatively equal distribution of PitNETs subtypes, sex and age. |
| 2 | Cheng,  et al. (2019) | Study design:  Case-control    surgery era:  2007-2016    Follow-up:  unknown | Diagnoses:  Non-functioning PitNETs    Number of participants  68    Important exclusion criteria  Other PitNETs than gonadotroph PitNET and null-cell PitNET were excluded.    Age at surgery  Mean 50.28 (24-75 yeas)    Sex  35 males, 33 females     Cases  46 invasive PitNETs    Invasiveness:  tumour extension beyond the lateral tangent of the intra- and supra-cavernous internal carotid artery (Grade 3 and 4) as defined by Knosp.    Controls:  22 non-invasive PitNETs | Tissue sampling method  frozen in liquid nitrogen and stored.    Method  Illumina Infinium MethylationEPIC 850K BeadChip    Methylation analysis, n samples  68    Ki-analysis presence  No    Methylation analysis control  NA | Outcomes:  Mean overall DNA methylation beta value  Differentially methylated probes  DMGs  Differently expressed genes    Results:  A total of 8,842 probes showing significant changes with \|Δβ\| > 0.1 between the invasive and non-invasive groups were included in the downstream analyses.    The heatmap shows the methylation status of all significant probes based on the β-values of these CpG sites across all 68 samples (p < 0.05 and \|Δβ\| > 0.1).    Increased methylation was observed with decreased expression of PHYHD1, LTBR, C22orf42, PRR5, ANKDD1A, RAB13, CAMKV, KIFC3, WNT4, and STAT6. Decreased methylation was observed with increased expression of MYBPHL. | Strengths:  - Combining DNA methylation and mRNA expression profile analyses  - AMKV, KIFC3, WNT4, and STAT6; decreased methylation; and increased MYBPHL expression. The methylation status and expression levels of these genes were validated by pyrosequencing and RT-PCR.    Limitations:  - Small sample size of the controls (n = 22).  - No multivariable predictive analysis    Risk of bias:  A. Selection bias:  Intermediate risk due to limited information about the selection procedure and inclusion and exclusion criteria.    B. Attrition bias:  Low risk due to complete follow-up.    C. Detection/information bias:  Intermediate risk. No mention of blinding the outcome assessor for invasiveness of the PitNETs or other patient factors. However, a standardized protocol for methylation analysis of PitNETs was performed. Differential information bias is less likely due to the clear definition of tumour invasiveness.    D. Confounding:  Intermediate: In the supplementary materials, a baseline table indicates a relatively equal distribution of PitNETs. However, sex was not equally distributed among invasive and non-invasive PitNETs. |
| 3 | Cheng,  et al. (2020) | Study design:  Case-control    Surgery era:  2007-2016    Follow-up:  Mean 64.9 moths (range 17-26 months) | Diagnoses:  Non-functioning PitNETs   (Null cell and gonadotroph).    Number of participants  71    Important exclusion criteria  Not mentioned  Age at surgery  Median 45 years, (range 14-72)    Sex  36 females, 9 males    Cases  21 NF-PitNETs with regrowth    Regrowth:  maximum tumour diameter increases more than 2 mm on enhanced MRI from the day of surgery to follow-up endpoint with or without the reappearance of visual disturbance, headache or hypopituitarism    Controls:  50 NF-PitNETs with no-regrowth | Tissue sampling method  immediately frozen in liquid nitrogen, for storage at −196 ◦C.  Method  Illumina Infinium MethylationEPIC 850K BeadChip    Methylation analysis, n samples  71    Ki-analysis  no    Methylation analysis controls.  NA | Outcomes:  Differentially methylated probes  Differentially methylated  genes  MicroRNA expression of differentially methylated  genes    Results:  1.There are 1,663 CpG sites in the promoter region related to 3,329 different genes that show significant differences in promoter DNA methylation status between regrowth and non-regrowth patients.    2. Of these genes, 2,788 were found to be hypomethylated and 541 were hypermethylated in the regrowth group.    3. Of the 501 genes, 139 are differentially promoter methylated and expressed.    4. A significant increase in DNA methylation levels was observed in the regrowth group compared to the non-regrowth group in FAM90A1, ETS2, STAT6, CYBRD1, and PYCARD. Decreased methylation levels were observed in MYT1L, ING2, KCNK1, and SH3GL2.    4. Pearson analyses showed a significantly negative correlation between the methylation status and expression levels of FAM90A1, MYT1L, ETS2, ING2, STAT6, KCNK1, PYCARD, and SH3GL2.     5. Patients who are younger (HR = 0.323, 95% CI = 0.121 to 0.863, p = 0.024), have decreased expression of FAM90A1 (HR = 0.233, 95% CI = 0.083 to 0.649, p = 0.005), and have increased expression of ING2 (HR = 3.020, 95% CI = 1.067 to 8.543, p = 0.037) are more likely to experience tumour regrowth. | Strengths:  - Multivariable Cox analysis of gene expression; however, there were only 21 regrowth events, indicating a statistical power similar to that of a two-variable model.  - Internal validation of candidate predictive biomarkers.  Limitations:  - No multivariable predictive analysis was performed with methylation status.  They performed a model with five variables, which is underpowered for the present study.    Risk of bias:  A. Selection bias:  Intermediate risk due to limited information about the selection procedure and inclusion and exclusion criteria.    B. Attrition bias:  Low risk. Complete follow-up. However, there are differences in follow-up time.    C. Detection/Information Bias:  Intermediate risk. There was no mention of blinding the outcome assessor to regrowth of PitNETs or other patient factors. The standardized protocol of methylation analysis for a pituitary PitNET was performed, however.    D. Confounding:  Intermediate: Multivariate Cox regression analysis including age. The supplementary materials present baseline tables indicating a relatively equal distribution of sex and type of pituitary PitNET. However, invasiveness (Knosp criteria) was unequal and could be an important confounding factor for progression-free survival (see Supplementary Table 1). |
| 4 | Garcia-Martinez, et al. (2019) | Study design:  Case-control    Surgery era:  unknown    Follow-up:  unknown | Diagnoses  PitNETs:   - 35 silent gonadotroph - 15 silent corticotroph - 15 functioning corticotroph - 40 functioning somatotroph     Number of participants  105    Important exclusion criteria:  Not mentioned    Cases  51 invasive PitNETs    Invasiveness: clinical manifestations, Hardy’s classification, and MRI invasiveness of the cavernous sinus      Controls  54 non-invasive PitNETs    Age at surgery  Mean 49.4 years (SD, 14.24).    Sex  45 males, 60 females | Tissue sampling method  Fresh frozen at -20°C.    Method  methylation-specific multiplex ligation-dependent probe amplification (MS-MLPA)  of 35 gene promotors.    Number of methylation analysis samples  105    Ki-analysis  No    Methylation analysis controls  4 reference probes that are not affected by HhaI digestion were included. | Outcomes:  DNA promotor methylation of 35 genes    Results:  ESR1 and RASSF1 exhibited higher levels of methylation in non-invasive tumours than in invasive tumours (P = 0.054 and P = 0.031, respectively).  The same trend was observed in the gonadotroph subtype (P = 0.055 and P = 0.050, respectively).  MacroPitNETs exhibited a higher prevalence of promoter methylation of the MSH6 and CADM1 genes than microPitNETs (P = 0.008 and P = 0.049, respectively). | Strengths:    Limitations:  - Unknown follow-up time  - No report of presurgical treatment dosages  - There is no clear definition of invasiveness (e.g., cavernous sinus invasion).    Risk of bias:  A. Selection bias:  High risk due to limited information about the selection procedure and inclusion/exclusion criteria. The place or time of recruitment is not mentioned.    B. Attrition bias:  Low risk. Complete follow-up.    C. Detection/Information Bias:  Intermediate risk. There was no mention of blinding the outcome assessor regarding invasiveness or PitNET subtype. However, a standardized protocol for the methylation analysis of PitNETs was performed.    D. Confounding:  Intermediate: No baseline table was presented for the invasive and non-invasive subgroups. Although a multivariable analysis was not performed, the influence of important clinical factors on methylation status was investigated. In the overall series, no correlation was found between the methylation status of the 35 analysed genes and participant sex, age, or tumour maximum diameter. |
| 5 | Gu,  et al (2016) | Study design:  Case-control    Surgery era:  unknown    Follow-up:  unknow | Diagnoses:  Non-functioning PitNETs    Important exclusion criteria  Not mentioned    Number of participants  12    Age at surgery  Mean age 49.1 years in the invasive and 56.4 in the non-invasive group    Sex  8 males, 11 females    Cases  10 invasive PitNETs    Invasiveness: Wilson-Hardy grading system: Grades III, IV, and V and Grades D and E    Controls:  9 non-invasive PitNETs | Tissue sampling method  fresh-frozen in liquid nitrogen and stored at -80 C    Method  Infinium Human Methylation450 BeadChip Arrays (450 k, Illumina).    Methylation analysis, n samples  19    Ki-analysis presence  yes    Methylation controls  Not mentioned | Outcomes:  Methylation level of CpGs    Results:  A total of 5,931 CpGs were selected as differentially methylated sites (DMSs) based on delta >0.15 and p value <0.01. There were significantly more hypomethylated DMSs than hypermethylated sites in the invasive PitNETs.    Cluster analysis of 339 CpGs (Delta >0.25 and p value <0.001) demonstrated complete distinction between invasive and non-invasive nonfunctioning groups.    GALNT9 mRNA expression, which contains a validated DMS, was significantly downregulated in the invasive group. However, there was no correlation between GALNT9 methylation and gene expression. | Strengths:  - Detailed definition of invasiveness    Limitations:  - Very limited sample size and statistically underpowered study.    Risk of bias:  A. Selection bias:  High risk due to limited information about the selection procedure and inclusion and exclusion criteria. The place or time of recruitment was not mentioned.    B. Attrition bias:  Low risk. Complete follow-up.    C. Detection/information bias:  Intermediate risk. There was no mention of blinding the outcome assessor for invasiveness or PitNET  subtype. However, a standardized protocol for methylation analysis of PitNETs was performed.    D. Confounding:  Intermediate: The baseline table showed an equal distribution of PitNET subtypes and sex. More female patients were present in the invasive group. No multivariable analysis was performed due to insufficient statistical power. |
| 6 | Guaraldi, et al. (2022) | Study design:  Case-control    Surgery era:  2003-2020    Follow-up:  8 years | Diagnoses:  PitNETs:   - Corticotroph: 25 - Gonadotroph: 50 - Lactotroph: 14 - Somatotroph: 19 - Null cell: 1 - Plurihormonal Pit1+: 2   Number of participants  111 participants    Important exclusion criteria   - No history of prior surgery or radiotherapy - No evidence of residual tumour on MRI 3 months - post-operative- follow-up at   least 1 year.  Age at surgery  Mean 51.5  years, (SD: 15.7) (26-87 years)    Sex  53 females, 57 males    Cases  41 aggressive PitNETs    6 pituitary carcinomas: presence of craniospinal and/or systemic metastases    Aggressiveness:  size >10 mm, extra‐sellar invasion (defined by Knosp grades 3–4/Hardy‐Wilson stages D–E), high proliferation (ki 67 ≥3% and p53 > 10 strongly positive nuclei/10 HPF or the presence of >2/ 10 mitoses/HPF), and requiring multiple treatment to obtain disease remission or, at least, control. (ESE 2018 guidelines)    Controls  64 non-aggressive PitNETs | Tissue sampling method  FFPE    Method  Targeted bisulfite next-generation sequencing (NGS) of 22 genes:  16 target genes mapped on autosomes (i.e., MIR137HG, LRRTM1, ZAP70, ITGA4, KIF1A, PDCD1, PARP15, TERT, Linc00599, AIP, FLI1, NTM, CDH1, MIR193a, GNAS, GP1BB), and 6 genes mapped on chromosome X (i.e., UXT, HDAC6, MAGEC2, MAGEA11, MAGEA1, FLNA)      Methylation analysis, number of samples  111, however not clearly mentioned    Ki-analysis presence  No    Controls for methylation analysis  Ten normal pituitaries from autopsies | Outcomes:  Methylation status of  22 targeted genes      Results:  The methylation levels of PARP15, LINC00599, and ZAP70 were higher in aggressive PITNETs than in non-aggressive ones. According to the Kruskal–Wallis test, the mean values for informative CpGs were 0.21, 0.08, and 0.48, respectively, for aggressive PITNETs and 0.11, 0.03, and 0.40, respectively, for non-aggressive ones.  The levels of methylation of AIP, GNAS, and PDCD1 were significantly lower in aggressive PitNETs /PITNETs (mean values for informative CpGs: 0.01, 0.31, and 0.64, respectively) than in non-aggressive ones (mean values for informative CpGs: 0.18, 0.48, and 0.70, respectively;  according to the Kruskal–Wallis test with p < .05).    For X-linked genes, males presented a higher level of methylation of the FLNA, UXT, and MAGE family (MAGEA11, MAGEA1, and MAGEC2) genes in aggressive versus non-aggressive PITNETs (p < .05). | Strengths:  - Testing methylation analysis of different tissue sampling methods    Limitations:  - No multivariable analysis was performed to account for important confounders.    Risk of bias:  A. Selection bias:  Intermediate risk due to limited information about the selection procedure and source population.      B. Attrition bias:  Low risk. Complete follow-up, and no difference in follow-up time between cases and controls.    C. Detection/information bias:  Intermediate risk. There was no mention of blinding the outcome assessor to the aggressiveness of PitNETs or other patient factors. However, a standardized protocol for methylation analysis of PitNETs was performed.    D. Confounding:  High risk: Baseline characteristics for cases and controls regarding sex and age at surgery are similar. However, PitNET size, pre-surgical medical therapy, and tumour type were significantly different between cases and controls and can be important confounders of aggressiveness. These factors should be accounted for in the statistical analysis. |
| 7 | Hallen, et al. (2022) | Study design:  Case- control    surgery era:  1987-2014    Follow-up:  5 years | Diagnoses:  Non-functioning PitNETs    number of participants:  43    Important exclusion criteria:   - Reintervention of a stable tumour remnant - Postoperative radiotherapy as part of the primary treatment. - Documented postoperative tumour progression that does not require reintervention. - Patients with no residual tumour after primary surgery - Other nonfunctioning pituitary neuroendocrine tumours (PitNETs) other than gonadotroph ones. - Failed CpG fraction   Age at surgery  56 years in the reintervention group and 64 years in the radiologically stable group.    Sex  32 males and 13 females.    Cases  26 patient with reintervention (reoperation or radiotherapy) due to tumour progression    Controls  17 postoperative residual tumours without tumour progression for at least 5 years | Tissue sampling method  paraffin-embedded formalin-fixed    Method  Chip-based array with Infinium MethylationEPIC 850K BeadChip platform    Methylation analysis, number of samples  43    Ki-analysis presence  no    Methylation analysis controls  No | Outcomes:  DMPs  DMRs    Results:  Gene bodies (58% hypermethylated versus 42% hypomethylated) and the TSS1500 region (64% hypermethylated versus 36% hypomethylated) exhibited greater hypermethylation in the reintervention group.  These regions are involved in regulating gene expression. Similarly, the 3 hypomethylated DMPs with the lowest P values in the reintervention group compared with the radiologically stable group were cg06624032 (adjusted P = 5.15 × 10–4, delta beta 0.23) located on chromosome 7 (gene body of CPED1), cg02457623 (adjusted P = 5.15 × 10–4, delta beta 0.38) located on chromosome 1 (the 5’UTR of ATP2B4), and cg14424181 (adjusted P = 7.20 × 10–4, delta beta 0.25) located on chromosome 20 (intergenic) | Strengths:  - Gene ontology analyses    Limitations:  Two outcomes in one: tumour progression and reintervention.    Risk of bias:  A. Selection bias:  Low risk. The selection procedure and inclusion and exclusion criteria are well described. However, excluding tumours that progress without the need for reintervention may select for the most aggressive PitNETs at baseline and overestimate the methylation results. If you compare the results with those of the general population of patients with re-growth after surgery,    B. Attrition bias:  Low risk due to strict exclusion criteria and complete follow-up.    C. Detection/information bias:  Moderate risk due to no mention of blinding the outcome assessor to reintervention status. However, a standardized protocol for methylation analysis of PitNETs was performed. Differential information bias is less likely due to the clear definition of methylation status and reintervention.      D. Confounding:  A low-risk, well-described baseline table was presented in the article. Important confounders: However, the mean age in the reintervention group was lower than in the radiologically stable group (56 vs. 64 years; P = 0.018). There were no differences between the patient groups with respect to preoperative tumour volume or preoperative hormone deficiency. |
| 8 | Jotanovic, et al. (2025) | Study design:  Case-control    surgery era:  not reported    Follow-up:  Not reported | Diagnoses:  PitNETs:   - Corticotroph: 33 - Gonadotroph: 6 - Lactotroph: 16 - Somatotroph: 9 - Somatolactotroph: 3 - Thyrotroph: 2 - Silent PIT1+: 4 - Plurihormonal: 3     number of participants:  76    Important exclusion criteria:   - Not reported     Cases  48 aggressive PitNETs (APT)    Aggressiveness: invasive tumour with unusually fast growth and/or clinically significant tumour progression despite surgery, radiotherapy, and standard medical therapy.   1. 16 pituitary carcinoma   Definition: pituitary tumours with presence of metastatic spread to the central nervous system and/or to distant locations    Controls  23 non-aggressive PitNETs    Age at diagnosis  Not reported    Sex  45 males, 31 females. | Tissue sampling method  FFPE    Method  Illumina Infini umMethylation EPIC (850 k) BeadChip Kit    Methylation analysis, number of samples  76    Ki-analysis presence  No  Methylation analysis controls  No | Outcomes:  - Differential methylation positions (DMPs)    Results:   Unsupervised hierarchical clustering based on the top 5000 most variable CpG sites revealed complete separation of the APT/ PC group from the benign tumour group.  A total of 9066 significant DMPs were detected with a p value < 1.3 × 10–7 and an absolute change in β value ≥ 0.2, when APT/PC and benign tumours were compared. Among APT/PCs, 7394 DMPs exhibited hyper methylation, whereas 1672 DMPs displayed hypomethylation. | Strengths:   - Use of ESE definition of aggressive pituitary tumours.   Limitation:   - No further analysis of differential methylated genes and gene expression.   Risk of bias:  A. Selection bias:  Low risk due to sufficient information about the selection procedure and source population.    B. Attrition bias:  Low risk due to complete follow-up.    C. Detection/information bias:  Moderate risk; no mention of blinding the outcome assessor to invasiveness status. However, a standardized protocol for methylation analysis of PitNETs was performed. Differential information bias is less likely due to the clear definition of aggressiveness.      D. Confounding:  Intermediate risk: Important confounders such as age, tumour size were not reported separately for aggressive and non-aggressive PitNETs. |
| 9 | Kayacan, et al. (2025) | Study design:  Case-control    surgery era:  2004-2017    Follow-up:  At least 3 months | Diagnoses:  Corticotroph PitNETs    number of participants:  32    Important exclusion criteria:   - No follow-up for at least 3 months in the outpatient clinic - Patients who did not provide adequate tissue samples for molecular analysis       Cases  9 invasive PitNETs    Invasiveness: Histologically confirmed invasion of the  dura or bone, or as per magnetic resonance imaging (MRI) indicating with Knosp grades 3 and 4 for cavernous sinus invasion.    Controls  23 non-invasive PitNETs    Age at diagnosis  Mean 37.31 years, SD 12.89    Sex  24 Females, 8 males | Tissue sampling method  FFPE    Method  MS-PCR    Methylation analysis, number of samples  21    Ki-analysis presence  yes    Methylation analysis controls  No | Outcomes:  - Methylation levels of the CDKN2A gene.    Results:  There was no difference in methylation status (partial vs no methylation) between invasive and non-invasive tumours (partial methylation: 50% vs 47.0% respectively).    Invasion status did correlation with CDKN2A gene expression. | Strengths:  - Validation procedure including analysis of DNA methylation patterns at selected gene promoters  - Validation procedure including gene expression analysis.    Limitation:   - Very small sample size for methylation analysis.   Risk of bias:  A. Selection bias:  Low risk due to sufficient information about the selection procedure and source population.    B. Attrition bias:  Low risk due to complete follow-up.    C. Detection/information bias:  Moderate risk; no mention of blinding the outcome assessor to invasiveness status. However, a standardized protocol for methylation analysis of PitNETs was performed. Differential information bias is less likely due to the clear definition of invasiveness.      D. Confounding:  Intermediate risk: Important confounders such as sex and age were not reported separately for invasive and non-invasive PitNETs. Additionally, a multivariable model or stratified statistical analysis for tumour subtype and age are missing. |
| 10 | Kober, et al. (2018) | Study design:  Case- control    surgery era:  not mentioned    Follow-up:  Not mentioned | Diagnoses:  Non-functioning PitNETs.    number of participants:  75    Important exclusion criteria:  Not mentioned      Age at surgery  Whole study population, n=75, median 60 years (34-85)  Genome wide DNA methylation n=34: median 60.5 years (36-85)    Sex  Whole study population, n=75, 49 males, 26 females.  Genome wide DNA methylation n=34: 22 males, 12 females.  Cases  18 invasive NF-PitNETs    Invasiveness: Tumour invasion was evaluated on preoperative MRIs for all patients, and the main directions of invasive growth were assessed. Cavernous sinus invasion was defined as the extension of a PitNET beyond the line corresponding to the lateral tangents of the intracavernous carotid artery (grades 3 and 4), as defined by Knosp et al. (1993; Micko et al., 2015). The infrasellar direction of invasive growth was considered if the bone and dura of the sellar floor, the sphenoid sinus, and the clivus were invaded. Suprasellar expansion was considered invasive only if leptomeningeal infiltration was observed. The invasiveness of PitNETs was considered only if confirmed preoperatively by the surgeon through endoscopic inspection of infiltrated areas and/or histology.    Controls  16 non-invasive NF-PitNETs | Tissue sampling method  Immediately frozen in liquid nitrogen, for storage at-80 C    Method  The Infinium HumanMethylation450 BeadChip (HM450K) (Illumina) and pyrosequencing assay.    Methylation analysis, number of samples  43 genome-wide analysis,  75 DNA pyrosequencing    Ki-analysis presence  no    Methylation analysis controls  6 normal pituitary samples from autopsy | Outcomes:  - DMPs  - DMRs  - Methylation levels of the IRKB and CNSKR1 genes.    Results:    Genome-wide methylation analysis  A slight decrease in the average DNA methylation level was observed for invasive NF-PitNETs compared to non-invasive ones, but the difference was insignificant (mean values of 0.5115 and 0.4873, respectively, with a p-value of 0.055).    Comparing DNA methylation in invasive (n = 18) versus non-invasive (n = 16) NF-PitNETs was unable to identify any statistically significant DMPs when applying the basic criteria of an average delta beta value (Db) greater than 0.3 and an adjusted p-value less than 0.05.  We relaxed these criteria to p<0.001 and delta beta of at least 0.2.  Interestingly, three probes representing   The promoter region of the ITPKB gene at the CGI shore (cg11401257, cg19160520, and cg11222527) was identified.    ITPKN and CNSKR1 methylation analysis  A significantly lower DNA methylation level of the ITPKB promoter was observed in invasive versus non-invasive pituitary neuroendocrine tumours (PitNETs) (28.2% versus 39%, respectively, p = 0.0306), as previously indicated by the results of the HM450K array.    A higher level of CNKSR1 DNA methylation was observed in invasive NF-PitNETs than in non-invasive NF-PitNETs (mean 51.65% vs. 39%, respectively, p = 0.0427).    The highest correlation coefficient was found for the CNKSR1 gene, whose promoter demonstrated increased DNA methylation in invasive NF-PitNETs . Accordingly, significantly lower CNKSR1 expression (fold change [FC] = 1.6) was observed in invasive versus non-invasive tumours, reflecting the DNA methylation status. | Strengths:  - Validation procedure including analysis of DNA methylation patterns at selected gene promoters  - Validation procedure including gene expression analysis.    Risk of bias:  A. Selection bias:  Intermediate risk due to limited information about the selection procedure and source population.    B. Attrition bias:  Low risk due to complete follow-up.    C. Detection/information bias:  Moderate risk; no mention of blinding the outcome assessor to invasiveness status. However, a standardized protocol for methylation analysis of PitNETs was performed. Differential information bias is less likely due to the clear definition of invasiveness.      D. Confounding:  Intermediate risk: In Supplementary Table 1, the baseline characteristics, such as the mean, are equal between the cases and controls. However, females are overrepresented in the invasive group (13/31) versus 11/42 in the non-invasive group. Additionally, a multivariable model or stratified statistical analysis for tumour subtype and age are missing. |
| 11 | Kochling,  et al (2016) | Study design:  Retrospective cohort study    Surgery era:  2009-2013    Follow-up:  Available or 77/85 (91%) of the patients.    Median 22 moths (range 0- 215 months) | Diagnoses:  85 primary and 15 recurrent PitNETs:   - Lactotroph: 11 - Corticotroph:18 - Somatotroph :10 - Gonadotroph:29 - Thyrotroph: 1 - Plurihormonal: 10 - Null cell : 21     Number of participants  100    Important exclusion criteria  Not mentioned    Age at surgery:  Median 53 years    Sex  48 females (48%) and 52 males (52%) | Tissue sampling method  FFPE    Method  MS-PCR of TERT promotor    Methylation analysis, number of samples  100    Ki-analysis presence  Yes    Methylation analysis controls  Yes, Specimen of FFPE brain tissue (negative control) | Outcomes:  -Tumour recurrence  - Progression free survival      Results:  -In grade of resection-based subgroup analyses, both tumour recurrence and PFS were similar comparing methylated and non-methylated individuals (p = n.s.).  Subsequently, rates of promoter methylation in primary and recurrent PitNETs were compared by uni- and multivariate analyses. Thus, promoter methylation was found in five of 15 samples of recurrent tumours (33%) and was therefore similar to methylation rates detected in primary diagnosed PitNETs (27%; n.s.). | Strengths:  - Presence of a control group for methylation analysis  - Tissue samples obtained at the initial surgery were available for each of the 13 recurrent tumours.    Risk of bias  A. Selection bias:  Intermediate risk due to limited information about the selection procedure and source population.    B. Attrition bias:  Low risk. There was 9% loss to follow-up, and it was non-selective.    C. Detection/information bias:  Intermediate risk. There was no mention of blinding the outcome assessor for progression-free survival, tumour recurrence, or other patient factors in PitNETs. However, a standardized protocol for methylation analysis of PitNETs was performed.    D. Confounding:  Intermediate risk: The association between methylation and tumour recurrence and progression-free survival was adjusted for resection grade. Important confounders such as sex and pituitary neuroendocrine tumour (PitNET) size were not included in the progression-free survival analysis. The analysis of the association between methylation status and tumour recurrence was adjusted for sex, pituitary neuroendocrine tumour (PitNET) size, and pathological PitNET subtype. However, due to the small number of recurrent PitNETs (13), there is only statistical power for univariate analysis, making the presence of a multivariable analysis less likely in this specific case. |
| 12 | Ling, et al. (2014) | Study design:  Case-control    Surgery era:  unknown    Follow-up:  unknown | Diagnoses:  PitNETs:   - Non-functioning: 17 - Somatotroph: 5 - Corticotroph: 1 - Silent corticotroph: 1   Number of participants  24    Important exclusion criteria  Not mentioned    Age at surgery  Mean 50 years, (range 16-92 years)    Sex  13 females an 11 males.    Cases  12 invasive PitNETs    Invasiveness: PitNETs with Knosp scores of 0–1 were classified as non-invasive, and those with Knosp scores of 2–4 were classified as invasive    Controls:  12 non-invasive PitNETs | Tissue sampling method   fresh-frozen in liquid nitrogen and stored at -80 ^0^ C until used for DNA and RNA extraction.    Method  Illumina Infinium HumanMethylation 450 (HM450) Beadchip platform     Methylation analysis, number of samples  24    Ki-analysis presence  No    Methylation analysis control  NA | Outcomes:  Mean DNA methylation beta value  DNA DMP, DMR and DMG.    Results:  No significant differences in global DNA methylation were found between invasive and non-invasive PitNETs, and tumour grade did not influence DNA methylation levels.  However, 34 CpGs (associated with 17 genes) were identified as hypomethylated in invasive NF-PitNETs compared to non-invasive ones. These CpGs were linked to enhancer regions and included genes such as FLT1 and SLIT3, which are involved in cell motility and invasion.  Attempts to validate methylation in these genes were unsuccessful due to probe limitations, and no methylation differences were observed in promoter regions between the two groups. | Limitations:  - Invasiveness is only defined as sinus cavernous invasion.    Risk of bias:  A. Selection bias:  Intermediate risk due to limited information about the selection procedure and source population. However, the methylation status was unknown at patient inclusion.    B. Attrition bias:  Low risk due to complete follow-up.    C. Detection/information bias:  Intermediate risk; no mention of blinding the outcome assessor to the invasiveness of the PitNETs or other patient factors. However, a standardized protocol for methylation analysis of PitNETs was performed.    D. Confounding:  Intermediate risk: The association between methylation and tumour was not adjusted for confounders, such as age and sex. Nevertheless, both groups appeared similar in terms of baseline characteristics, such as age and sex. There was, however, diversity in PitNET subtypes and differences in pituitary tumour size. Due to the small sample size, there was insufficient statistical power to perform a multivariable analysis. |
| 13 | Miyake, et al (2018) | Study design:  Retrospective cohort study    surgery era:  unknown    Follow-up:  unknown | Diagnoses:  PitNETs:   - Non-functioning: 59 - Somatotroph: 8 - Corticotroph: 1 - Lactotroph: 1 - Thyrotroph: 1     number of participants:  70    Important exclusion criteria:  Not mentioned    Age at surgery  Median 59 years (range 22-81 years)    Sex  Males 43 (61%), 58 (83%) | Tissue sampling method  Fresh frozen (56/70 samples) or paraffin-embedded formalin-fixed (14/70 samples)    Method  methylation-sensitive high-resolution melting analysis (MS-HRM) of TERT gene.    Methylation analysis, number of samples  70    Ki-analysis presence  Yes    Methylation analysis controls  Yes, as positive (100% methylated) and negative (0% methylated) controls, we used CpGenome™ Universal Methylated and Unmethylated DNA (Chemicon, Millipore, Billerica, MA, USA), respectively | Outcomes:  Progression-free survival (PFS): Calculated from the date of surgery until disease progression is confirmed by neuroimaging.    Disease progression was defined as (1) a 30% increase in tumour volume, (2) a 10% increase in any dimension following incomplete resection, or (3) any detectable disease following complete resection.    TERT mRNA expression      Results:  A higher frequency of TERT promotor methylation was observed in the recurrent PitNETs (41.2%) compared to primary PitNETs (7.5%).  Methylated PitNETs had shorter PFS (median: 30 vs. 133 months), with TERT promoter methylation associated with an increased hazard of reduced PFS (HR: 5.804, 95% CI: 1.407–23.940).    TERT mRNA expression levels were significantly higher in the methylated group than in the non-methylated group (p = 0.008). | Strengths:  Additional Analysis:  mRNA expression analysis    Risk of bias  A. Selection bias:  Intermediate risk due to limited information about the selection procedure and source population. However, the unknown methylation status at patient inclusion makes selection bias less likely.    B. Attrition bias:  Low risk due to complete follow-up.    C. Detection/information bias:  Intermediate risk; no mention of blinding the outcome assessor to progression-free survival or other patient factors. However, a standardized protocol for methylation analysis of PitNETs was performed. Differential information bias is less likely due to the clear definition of methylation status and progression-free survival.      D. Confounding:  Low risk for the association between methylation status and progression-free survival. A multivariable Cox proportional hazards analysis was performed, including important confounders such as age, sex, largest tumour dimension, resection rate, and tumour status. However, the baseline table already showed similar distributions of the other confounders, except for tumour status (primary or recurrent). One could argue that a multivariable analysis including tumour and methylation statuses should suffice. In this situation, one could overcorrect for confounding variables. |
| 14 | Møller, et al. (2024) | Study design:  Retrospective cohort study    Surgery era:  2007-2017    Follow-up:  Median 75 months (12-167) | Diagnoses:  Non-functioning macroPitNETs    Number of participants  42    Important exclusion criteria:   - Incomplete medical history before surgery - PitNET volume smaller than 3 cm3.     Age at surgery  61 years (26-87 years)    Sex  16 females (38%), 24 (62%) males | Tissue sampling method  formalin-fixed and paraffin-embedded (FFPE) tissue samples    Method  Illumina’s MethylationEPIC array    Methylation analysis, number of samples  42    Ki-analysis presence  No    Controls  na | Outcomes:  no regrowth, low regrowth rate, and high regrowth rate.    Results:  Among SF1-lineage NF-PitNETs , we identified five methylation clusters, each with differentially methylated sections corresponding to distinct intracellular signalling pathways. The clusters showed no statistically significant differences in accumulated regrowth percentages.    A mixed-effects model was used to compare accumulated regrowth over eight years (the median follow-up for the cohort was 75 months) among the five methylation clusters, and no statistically significant differences were observed. | Strengths:  A linear mixed-effects model was used to examine whether different DNA methylation clusters exhibited distinct regrowth patterns. The analysis accounted for preoperative tumour volume, age at first surgery, reoperation, and additional radiotherapy. The model included adjustments for reintervention.    Limitations:  - Very small methylation subgroup due to the small sample size, resulting in limited statistical power.    Risk of bias:  A. Selection bias:  Low risk due to well-described inclusion criteria. Additionally, the methylation status was unknown at the time of patient inclusion, which makes selection bias less likely.      B. Attrition bias:  Low risk due to complete follow-up.    C. Detection/information bias:  Intermediate risk. There was no mention of blinding the outcome assessor for regrowth, reintervention, or other patient factors. However, a standardized protocol for methylation analysis of a pituitary PitNET was performed. Differential information bias is less likely due to the clear definition of methylation status, regrowth rate, and reintervention.      D. Confounding:  Low risk for the association between methylation status and progression rate. A multivariable linear mixed-effects model was performed, including important confounders such as preoperative tumour volume, age at first surgery, reoperation, and additional radiotherapy. The baseline table for the different methylation pattern groups showed no difference in age at first surgery. There was also no difference in invasive growth, mean preoperative and postoperative tumour volume, or median follow-up time. |
| 15 | Qian, et al. (2007) | Study design:  Case-control    Surgery era:  unknown    Follow-up:  unknown | Diagnoses:  Clinically Functional PitNETs:   - Somatotroph:1 - Mammosomatotroph:2 - Lactotroph:12 - Corticotroph:4 - Thyrotroph:3    Clinically non-functioning PitNETs   - silent corticotroph:6 - gonadotroph:14 - subtype 3: - null cell:1 PitNET   Number of participants:  69 participants    Important exclusion criteria:  Not mentioned.    Cases:  33 invasive PitNETs    Invasiveness:  Preoperative radiological investigations, operative findings, and a modified Hardy's classification were used. Grade I tumours (microPitNETs, ≤1 cm in diameter) and grade II tumours (enclosed macroPitNETs, ≤1 cm in diameter, with or without suprasellar extension) are defined as non-invasive. Grade III tumours (local invasion of the sphenoid and/or cavernous sinus) and grade IV tumours (central nervous system/extracranial spread, with or without metastasis) were considered invasive.    Controls:  36 non-invasive PitNETs    Age at surgery:  Mean: 62.5 years (SD: 15.7; 26–87 years).    Sex:  36 females and 33 males | Tissue sampling method  Fresh frozen tissue samples    Method  MS-PCR CDH1 and CDH13 gene.      Methylation analysis, number of samples  69    Ki-analysis presence  No      Methylation analysis controls  Five normal human pituitaries | Outcomes:  Methylation status of CDH13 and CDH1 gene.      Results:  Methylation of CDH13 was observed more frequently in invasive PitNETs(42%) than in non-invasive PitNETs (19%) (P < 0.05). Additionally, methylation of CDH1 was more prevalent in grade IV PitNETs than in grade I PitNETs (P < 0.05).    CDH13 mRNA expression was significantly lower in invasive adenomas than in non-invasive adenomas P=0.037    The expression of E-cadherin was significantly lower in invasive (grades IV and III) and macro- (grade II) than in non-invasive micro- (grade I) tumours (P=0.01, P=0.029, and P=0.015, respectively; | Strengths:  Gene expression analysis    Limitations:    Risk of bias  A. Selection bias:  Intermediate risk due to limited information about the selection process and source population. However, since the methylation status was unknown at the time of patient enrolment, selection bias is less likely.  Additionally, the study is less likely to be generalizable to all clinical pituitary tumours since none of the examined tumours had evidence of postoperative recurrence, especially the invasive ones. This is probably due to the short follow-up period.    B. Attrition bias:  Low risk; complete follow-up.    C. Detection/information bias:  Intermediate risk; no mention of blinding the outcome assessors to invasiveness or other patient factors. However, a standardized protocol for methylation analysis of PitNETs was performed. Differential information bias is less likely due to the clear definition of methylation status and tumour invasiveness.      D. Confounding:  Intermediate risk. The baseline table showed that there are no significant differences in confounding variables, such as age and sex, for methylated and unmethylated CDH13 and CDH1 gene PitNETs. However, there are rather large differences in PitNET subtypes. No multivariable or stratified analysis of PitNET subtypes was performed. |
| 16 | Rusetska, N et al. (2021) | Study design:  Case-control    Surgery era:  2010-2016    Follow-up:  Median 71 months (50-122 months) | Diagnoses:  Non-functioning PitNET  (68 primary and 12 recurrent pituitary PitNET)    Number of participants  80    Important exclusion criteria   Not mentioned    Age at surgery  Median 59.5 years (34-82 years)    Sex  46 males. 34 females      Cases  54 Invasive PitNETs    Invasiveness  Cavernous sinus invasion was defined by extension of tumour beyond the line corresponding to the lateral tangents of the intracavernous carotid artery (Grade 3 and 4), as defined by Knosp. Infrasellar direction of invasive growth was considered if bone and dura of the sellar floor, sphenoid sinus and clivus were invaded. The suprasellar expansion was considered invasive only if leptomeningeal infiltration was observed. Invasiveness of pituitary tumours was taken into account when it was confirmed by the surgeon with endoscopic inspection of infiltrated areas and/or by histology.    Controls  26 non-invasive PitNETs | Tissue sampling method  immediately frozen in liquid nitrogen, for storage at −80 ◦C    Method  Genome-wide methylation analysis with Infinium HumanMethylation450 BeadChip (HM450K) (Illumina))    Analysis of Line-1 with pyrosequencing    Methylation analysis, number of samples  80    Ki-analysis  Yes, cut-off 3%    Methylation analysis controls:  Yes, Five samples of normal human pituitary tissue from autopsies were used | Outcomes:  Median beta methylation levels  LINE-1 methylation levels    Results:      Line-1  When PitNETs stratified according to tumour invasion status were compared, lower LINE-1 methylation levels were observed in invasive PitNETs than in non-invasive ones (mean value 68.07% vs. 71.45%, respectively; p = 0.0192)    The diagnostic value with area under the ROC Curve (AUC) was 0.663 (95% CI 0.5451 to 0.7802; p = 0.0139)    Expression levels of two open reading frames of LINE-1 retrotransposon (L1-ORF1 and L1-ORF2) in tumour samples included in the LINE-1 methylation were assessment. Unfortunately, when we compared the expression levels of L1-ORF1 and L1-ORF2 between invasive and non-invasive tumours, no significant difference was observed | **Notes**    Strengths:   - Line- 1 mRNA expression analysis - Line-1 protein expression analysis - KI-analysis     Limitations:    Risk of bias  A. Selection bias:  Intermediate risk, due to limited information about the selection procedure and inclusion and exclusion criteria.  However, methylation status was unknown at the time of patient enrolment, making selection bias less likely.    B. Attrition bias:  low risk, Complete follow-up.    C. Detection/information bias:  Intermediate risk, no mentioning of blinding of the outcome assessor for tumour invasiveness or  patient factors. However, a standardized protocol of methylation analysis for a pituitary PitNET was performed. Differential information bias is less likely due to the clear definition of the methylation status and tumour invasiveness.      D. Confounding  Intermediate risk, In the supplementary table baseline characteristics are presented. Age and sex are  equal distributed in the invasive and non-invasive group. Furthermore stratified methylation analysis for important confounders such as tumour size, proliferation index and tumour recurrence were performed. However a multivariable model is missing. |
| 17 | Tsegaye, et al. (2025) | Study design:  Case-control    Surgery era:  2010-2016    Follow-up:  Unknown | Diagnoses:  Gonadotroph PitNETs  Number of participants  47    Important exclusion criteria  Not mentioned    Age at surgery  27 males, 20 females    Sex  27 males, 20 females     Cases  22 invasive PitNETs    Invasiveness:  Knosp Grade 3 and 4 and if bone and dura of the sellar floor, sphenoid sinus, and clivus were invaded or leptomeningeal infiltration was observed.  Invasiveness of the tumour was taken into account only if it was preoperatively confirmed by the surgeon with endoscopic inspection of infiltrated areas and/or with histology.    Controls:  30 non-invasive PitNETs | Tissue sampling method  frozen in nitrogen liquid  Method  Pyrosequencing of e upstream promoter region of MIR184  Methylation analysis, number of samples  47    Ki-analysis presence  No    Methylation analysis controls  Pituitary tissue | Outcomes:  hsa-miR-184 promotor methylation status    Results:  No significant difference in hsa-miR-184 promotor methylation between invasive and non-invasive tumours.  Higher hsa-miR-184 level was observed in invasive than in non-invasive tumours (p = 0.0265).  No difference was observed in the relation between has-miR-184 levels and features of aggressive growth including Ki67 expression, newly diagnosed/recurrent tumour status, and tumour relapse during follow-up. | **Notes**    Strengths:   - Stratification analysis for aggressive growth, tumour size, ki67, and tumour status.     Limitations:  **Risk of bias**  A. Selection bias:  Intermediate risk, due to limited information about the selection procedure and inclusion and exclusion criteria. However, methylation status was unknown at the time of patient enrolment, making selection bias less likely.    B. Attrition bias:  low risk, Complete follow-up.    C. Detection/information bias:  Intermediate risk, no mentioning of blinding of the outcome assessor for the methylation cluster status other patient factors. Also no clear definition of tumour invasiveness was described in the article. However, the standardized protocol of methylation analysis for a pituitary PitNET was performed.    D. Confounding  Intermediate risk, no baseline characteristics were reported for invasive and non-invasive PitNETs separately. However a stratification analysis for important confounding factors such as aggressive growth, tumour size, ki67percentage, and newly diagnosed/recurrent tumour status was performed. |
| 18 | Valiulyte, et al. (2017) | Study design:  Case-control    Surgery era:  2010-2016    Follow-up:  Unknown | Diagnoses:  PitNETs:   - Non-functioning:8 - Somatotroph:6 - Lactotroph:36 - Corticotroph:1 - Plurihormonal:11   Number of participants  102 patients    Important exclusion criteria  Not mentioned      Age at surgery  < 60 years:  40 patients, >60 year 62 patients.    Sex  Female 58, male 44     Cases  58 invasiveness PitNETs    invasiveness:  magnetic resonance imaging findings and the Hardy classification, modified by Wilson was used to quantify the sphenoid sinus invasion and suprasellar extension    Controls:  30 non-invasive PitNETs | Tissue sampling method  frozen in nitrogen liquid    Method  Methylation-specific polymerase chain reaction (MS-PCR) of STAT3   Methylation analysis, number of samples  88 for invasiveness as outcome, in total 102    Ki-analysis presence  No    Methylation analysis controls  normal human blood lymphocyte DNA treated with bisulfite (non-methylated control), the Bisulfite-Converted Universal Methylated Human DNA Standard (Zymo Research, USA) (methylated control) and nuclease-free water (negative control). | Outcomes:  STAT3 gene methylation status    Results:  invasive PitNETs showed low methylation status of STAT3 gene. Only in 12.07% (7/ 58) invasive PitNETs STAT3 gene was methylated and in the non-invasive PITNETS It was 2/30 (6.67%) (χ2 test, p = 0.428)    There was no significant association between STAT3 mRNA expression and PitNET invasiveness (p=0.798). | **Notes**    Strengths:   - mRNA expression of STAT3     Limitations:   - No clear definition of tumour invasiveness.     **Risk of bias**  A. Selection bias:  Intermediate risk, due to limited information about the selection procedure and inclusion and exclusion criteria. However, methylation status was unknown at the time of patient enrollment, making selection bias less likely.    B. Attrition bias:  low risk, Complete follow-up.    C. Detection/information bias:  Intermediate risk, no mentioning of blinding of the outcome assessor for the methylation cluster status other patient factors. Also no clear definition of tumour invasiveness was described in the article. However, the standardized protocol of methylation analysis for a pituitary PitNET was performed.    D. Confounding  Intermediate risk, The baseline table showed that for methylated and unmethylated STAT3 gene PitNETs, there are no significant differences in confounding variables such as age groups and sex. However, there is a rather large difference in PitNET subtypes, and no multivariable or stratified analysis for PitNET subtypes was performed. |
| 19 | Yang, Y  et al. (2024) | Study design:  Case-control    Surgery era:  March 2020- December 2024    Follow-up:  unknown | Diagnoses:  Corticotroph PitNETs      Number of participants  40    Important exclusion criteria     - patients having undergone previous gamma knife radiotherapy or surgery; - having incomplete data; postoperative histopathological diagnosis of non-pituitary PitNET; - combined renal disease or cardiovascular or cerebrovascular disease; - or having uncooperative information after consultation.   Cases  20 Invasive PitNETs    Invasiveness: Invasive PA was defined as Knosp classification grades III–IV and HardyWilson classification grades III–IV [27,28]; non-invasive PA was defined as a tumour confined to the tissue without any compression of surrounding structures [29]    Controls:     1. 20 patients with non-invasive pituitary PitNET      1. normal pituitary tissues from the  20 cases     Age at surgery  Not mentioned    Sex  Not mentioned | Tissue sampling method  immediately frozen in liquid nitrogen, for storage at −80 ◦C    Method  MS-PCR TMIP1-3 gene.    Methylation analysis, number of samples  9 ( 3 cases, and 3 non-invasive controls and 3 normal pituitary tissue controls from cases)    Ki-analysis presence  No    Methylation analysis controls  Episcope® CpG methylase-treated HCT116 gDNA (Clontech, Mountain View, CA, USA) was used as the positive control, and distilled water was used as the negative control | Outcomes:  TIMP1-3 methylation status  TIMP1-3 mRNA expression  TIMP1-3 protein levels    Results:    TIMP1-3 showed downregulated expression in invasive PitNET tissues and cell lines (p < 0.05).    The low expression of TIMP1- 3 was due to promoter methylation of these genes (p < 0.05).    The MSP assay confirmed 100% methylation of TIMP2 (Fig. 1C) and TIMP3 (Fig. 1D) in three typical cases of invasive pituitary tumours, whereas 0% methylation was observed in non | Notes    Strengths:   - control pituitary tissue from the cases.     Limitations:   - very limited sample size for methylation analysis (N=6)     **Risk of bias**  A. Selection bias:  Intermediate risk, due to limited information about the selection procedure and inclusion and exclusion criteria. However, methylation status was unknown at the time of patient enrolment, making selection bias less likely.    B. Attrition bias:  low risk, Complete follow-up. However, very small sample size    C. Detection/information bias:  Intermediate risk, no mentioning of blinding of the outcome assessor for invasiveness or other patient factors.    D. Confounding  High risk, no baseline table presented and no multivariable analysis has been performed.    **This study is excluded after quality assessment.** |
| 20 | Yuan, et al. (2008) | Study design:  Case-control    Surgery era:  unknown    Follow-up:  unknown | Diagnoses:  33 clinically functioning tumours :   - somatotroph:17 - mammosomatotroph: 1 - 10 lactotroph:10 - corticotroph:4 - 1 thyrotroph      20 clinically nonfunctioning PitNETs:   - silent corticotroph:4 - gonadotroph:12 - silent subtype:3 - 1 null cell :1     Number of participants  53    Important exclusion criteria:  Not mentioned    Cases  26 invasive PitNETs    Invasiveness:  Tumour size and invasiveness were defined on the basis of preoperative radiological investigations and operative findings and with a modified Hardy’s classification. Grade I tumours are microPitNETs (> 1cm in diameter) and grade II tumours consisted of enclosed macroPitNETs (<1cm in diameter) with or without suprasellar extension. Both grade I and II tumours were defined as non-invasive. Grade III tumours show local invasiveness with evidence of bony destruction and tumour within the sphenoid and/or cavernous sinus. Grade IV tumours demonstrate CNS/extracranial spread with or without metastases. Grade III and IV tumours were considered to be invasive.    Controls  27 Non-invasive PitNETs    Sex  25 males and 28 females    Age at surgery  Not mentioned | Tissue sampling method   frozen at -20°C    Method  MS-PCR of GSTP-1    Methylation analysis, number of samples  53    Ki-analysis presence  yes    Methylation analysis Controls  3 normal pituitary tissue | Outcomes:  GSTP1 methylation    Results:  There was no significant correlation between GSTP1 methylation and patient age.    GSTP1 methylation was detected more frequently in grade II, III, and IV tumours (66.7, 85, and 83%, respectively) than in grade I tumours (33%, P < 0.05). In addition, the frequency of GSTP1 methylation was higher in invasive tumours (84.6%) than in non-invasive tumours (63%; P< 0.05, Table 5).    GSTP1 expression levels were significantly lower in pituitary tumours with GSTP1 methylation than in pituitary adenomas without GSTP1 methylation (Figure 3b, Po0.05). Overall, there was a significant correlation between GSTP1 promoter hypermethylation and reduced-GSTP1 expression.    The difference In frequency between tumour type and patient gender was not statistically significant  The Ki-67 LI was also not significantly related to GSTP1 methylation in PitNETs. | Notes    Strengths:   - Detailed definition of invasiveness - Ki-67 analysis - 3 normal pituitary tissue controls         **Risk of bias**  A.Selection bias:  Intermediate risk, due to limited information about the selection procedure and inclusion and exclusion criteria.    B. Attrition bias:  low risk, Complete follow-up.    C. Detection/information bias:  Intermediate risk, no mentioning of blinding of the outcome assessor for the methylation cluster status other patient factors. However, standardized protocol of methylation analysis for a pituitary PitNET was performed. Differential information bias is less likely due to the clear definition of tumour invasiveness.      D. Confounding  Intermediate risk, The baseline table showed that the male/female distribution for invasive and non-invasive PitNETs is more or less the same.  However, the tumour types are very different between cases and controls.  Although there was no significant correlation between GSTP1 methylation and patient age, sex, and tumour type, a potential confounding effect of these factors cannot be excluded based on the analysis. Multivariable analysis was not performed. |

Abbreviations: MS-PCR: Methylation-specific polymerase chain reaction, MS-HRM: methylation-sensitive high-resolution melting analysis, MS-MLPA: methylation-specific multiplex ligation-dependent probe amplification (MS-MLPA). RRBS: reduced representation bisulfite sequencing, DMP: Differentially methylated position. DMR: Differentially methylated region. DMG: Differentially methylated gene.

**S4. QUIPS – Quality in Prognosis Studies**

|  | Study participation | Study attrition | PF measurement | Outcome measurement | Study confounding | Statistical analysis and reporting |  |
| --- | --- | --- | --- | --- | --- | --- | --- |
| Chen 2025 | 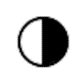 | 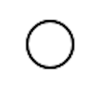 | 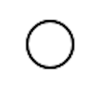 | 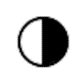 | 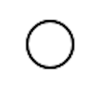 | 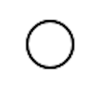 | 1/6 |
| Cheng 2019 | 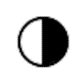 | 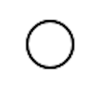 | 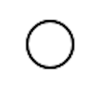 | 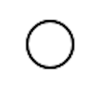 | 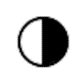 | 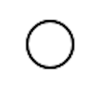 | 1/6 |
| Cheng 2020 | 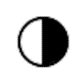 | 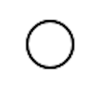 | 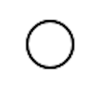 | 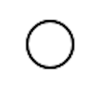 | 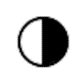 | 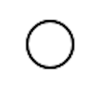 | 1/6 |
| Garçia- Martinez 2019 | 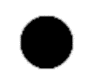 | 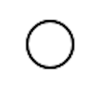 | 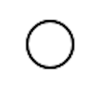 | 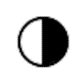 | 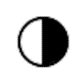 | 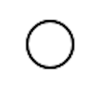 | 3/6 |
| Gu 2016 | 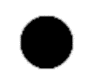 | 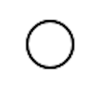 | 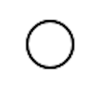 | 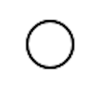 | 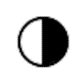 | 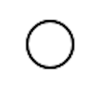 | 2/6 |
| Guaraldi 2022 | 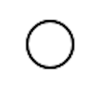 | 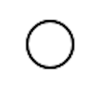 | 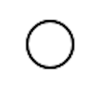 | 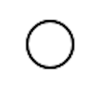 | 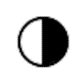 | 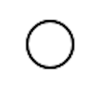 | 1/6 |
| Hallén 2022 | 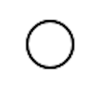 | 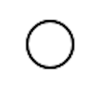 | 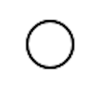 | 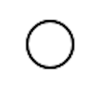 | 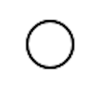 | 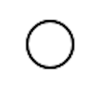 | 0/6 |
| Jotanovic 2025 | 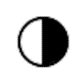 | 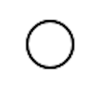 | 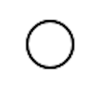 | 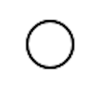 | 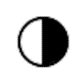 | 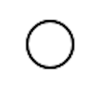 | 1/6 |
| Kayacan 2025 | 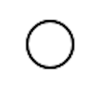 | 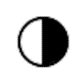 | 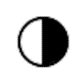 | 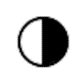 | 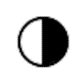 | 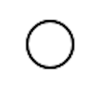 | 2/6 |
| Kuber 2018 | 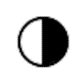 | 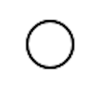 | 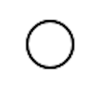 | 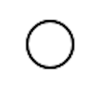 | 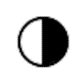 | 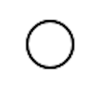 | 1/6 |
| Kochling 2016 | 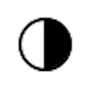 | 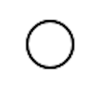 | 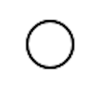 | 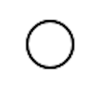 | 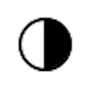 | 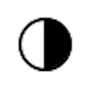 | 2/6 |
| Ling 2014 | 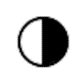 | 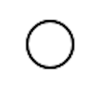 | 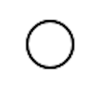 | 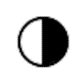 | 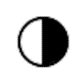 | 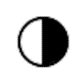 | 2/6 |
| Miyake 2018 | 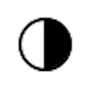 | 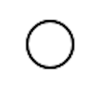 | 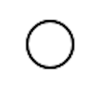 | 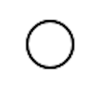 | 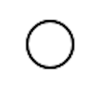 | 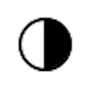 | 1/6 |
| Møller 2024 | 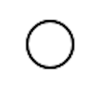 | 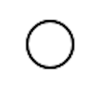 | 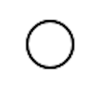 | 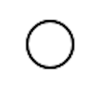 | 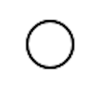 | 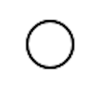 | 0/6 |
| Qian 2007 | 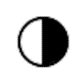 | 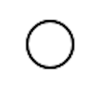 | 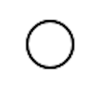 | 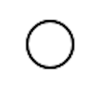 | 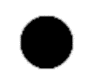 | 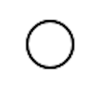 | 2/6 |
| Rusetska 2021 | 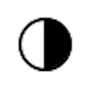 | 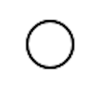 | 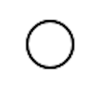 | 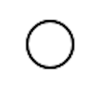 | 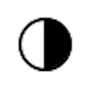 | 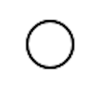 | 1/6 |
| Tsegaye 2025 | 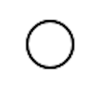 | 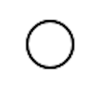 | 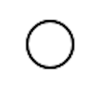 | 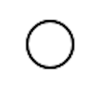 |  |  | 0/6 |
| Valiulyte 2017 |  |  |  |  |  |  | 2/6 |
| Yang 2024 |  |  |  |  |  |  | 4/6 |
| Yuan 2008 |  |  |  |  |  |  | 2/6 |

Low risk of bias RRBS

High risk of bias

Moderate risk of bias

Red 4-6: low quality

Orange 2-3: moderate quality

Green 0-1: high qualit
